# Targeting senescence-like fibroblasts radiosensitizes non–small cell lung cancer and reduces radiation-induced pulmonary fibrosis

**DOI:** 10.1172/jci.insight.146334

**Published:** 2021-12-08

**Authors:** Jingshu Meng, Yan Li, Chao Wan, Yajie Sun, Xiaomeng Dai, Jing Huang, Yan Hu, Yanan Gao, Bian Wu, Zhanjie Zhang, Ke Jiang, Shuangbing Xu, Jonathan F. Lovell, Yu Hu, Gang Wu, Honglin Jin, Kunyu Yang

**Affiliations:** 1 Cancer Center and; 2 Department of Thoracic Surgery, Union Hospital, Tongji Medical College, Huazhong University of Science and Technology, Wuhan, China.; 3 Department of Chemical and Biological Engineering, University at Buffalo, State University of New York, Buffalo, New York, USA.; 4 Institute of Hematology, Union Hospital, Tongji Medical College, Huazhong University of Science and Technology, Wuhan, China.

**Keywords:** Oncology, Cellular senescence, Fibrosis, Radiation therapy

## Abstract

Cancer cell radioresistance is the primary cause of the decreased curability of non–small cell lung cancer (NSCLC) observed in patients receiving definitive radiotherapy (RT). Following RT, a set of microenvironmental stress responses is triggered, including cell senescence. However, cell senescence is often ignored in designing effective strategies to resolve cancer cell radioresistance. Herein, we identify the senescence-like characteristics of cancer-associated fibroblasts (CAFs) after RT and clarify the formidable ability of senescence-like CAFs in promoting NSCLC cell proliferation and radioresistance through the JAK/STAT pathway. Specific induction of senescence-like CAF apoptosis using FOXO4-DRI, a FOXO4-p53–interfering peptide, resulted in remarkable effects on radiosensitizing NSCLC cells in vitro and in vivo. In addition, in this study, we also uncovered an obvious therapeutic effect of FOXO4-DRI on alleviating radiation-induced pulmonary fibrosis (RIPF) by targeting senescence-like fibroblasts in vivo. In conclusion, by targeting senescence, we offer a strategy that simultaneously decreases radioresistance of NSCLC and the incidence of RIPF.

## Graphical abstract

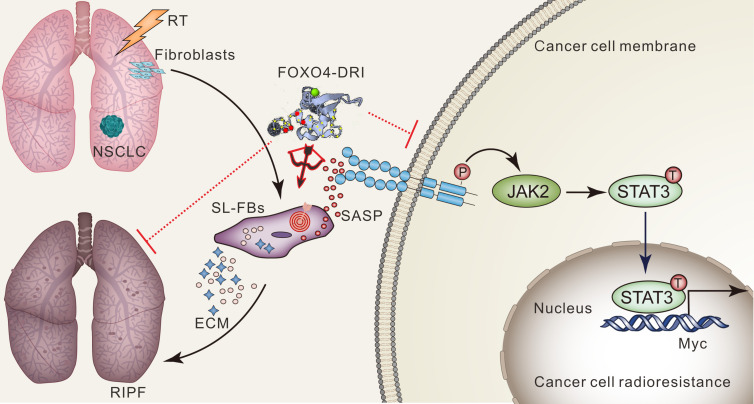

## Introduction

Non–small cell lung cancer (NSCLC), accounting for about 85% of newly diagnosed lung cancer cases, is the leading cause of cancer-related mortality worldwide (1, 2). Despite the notion that radiotherapy (RT) plays a critical role in both curative and palliative treatments of NSCLC, there remains a group of patients suffering from temporary remission or rapid progression due to radioresistance (3). Although cancer cell radioresistance could be overcome by increasing the RT doses applied, the maximum radiation dose is often limited by normal tissue tolerance. Optimized dose delivery and fractionation could help decrease normal tissue injury, allowing for a higher RT dose application, but the effects are usually not sufficient to overcome cancer cell radioresistance (3). Chemotherapeutic agents, small-molecule inhibitors, antibodies, or nanomaterials are frequently used as radiosensitizers, although only limited success has been achieved as tumor microenvironment (TME) changes are often ignored (4, 5). In fact, alterations in TMEs induced by RT — including inflammation, revascularization, cycling hypoxia, immunomodulation, and extracellular matrix (ECM) remodeling — play a pivotal role in RT responses (5, 6). As abundant stromal cells in the TME, cancer-associated fibroblasts (CAFs) protect cancer cells from radiation-induced death and increase cancer cell radioresistance via paracrine action by cytokines, such as CXCL1, CXCL12, IGF1/2, and PDGF, eventually leading to RT failure and cancer progression (5, 7–9). To the best of our knowledge, the combination of targeting CAFs and RT for cancer therapy has not been reported. Furthermore, because there is a lack of specific targeting biomarkers due to the heterogeneity of fibroblasts in TMEs, effective strategies to target whole CAF populations are also not available yet. Consequently, novel strategies of targeting CAFs for radiosensitization should be of great interest.

It has been shown that senescence in TMEs — especially in stromal cells — is a primary cause of therapy resistance and cancer relapse (10–13). Abundant evidence indicates that senescence-like fibroblasts can promote tumorigenesis and tumor growth in various malignancies, via paracrine action in a senescence-associated secretory phenotype (SASP), wherein cells secrete a wide range of inflammatory cytokines, chemokines, growth factors, and matrix remodeling enzymes (14–20). In addition, senescence-like fibroblasts have been reported to participate in the pathogenesis of pulmonary fibrosis (PF), indicating further relevance for the detection of established senescence-related fibroblast biomarkers, such as CDKN2A, CDKN1A, and senescence-associated β-galactosidase (SA–β-Gal) (21–23). Because senescent cells can be targeted by senolytic drugs, radiation-induced senescence-like fibroblasts may be developed as a promising target for decreasing radioresistance and alleviating radiation-induced pulmonary fibrosis (RIPF).

A cell-penetrating peptide (FOXO4-DRI), which perturbs the FOXO4 interaction with p53, can selectively cause p53 nuclear exclusion and cell-intrinsic apoptosis in senescent cells (24). In this study, we explored whether targeting radiation-induced senescence-like fibroblasts with FOXO4-DRI could have a “1 stone, 2 birds” effect — i.e., to decrease NSCLC cells’ radioresistance and reduce RIPF.

## Results

### Senescence-like CAFs promote NSCLC cell proliferation and radioresistance in vitro.

**Figure 1:**
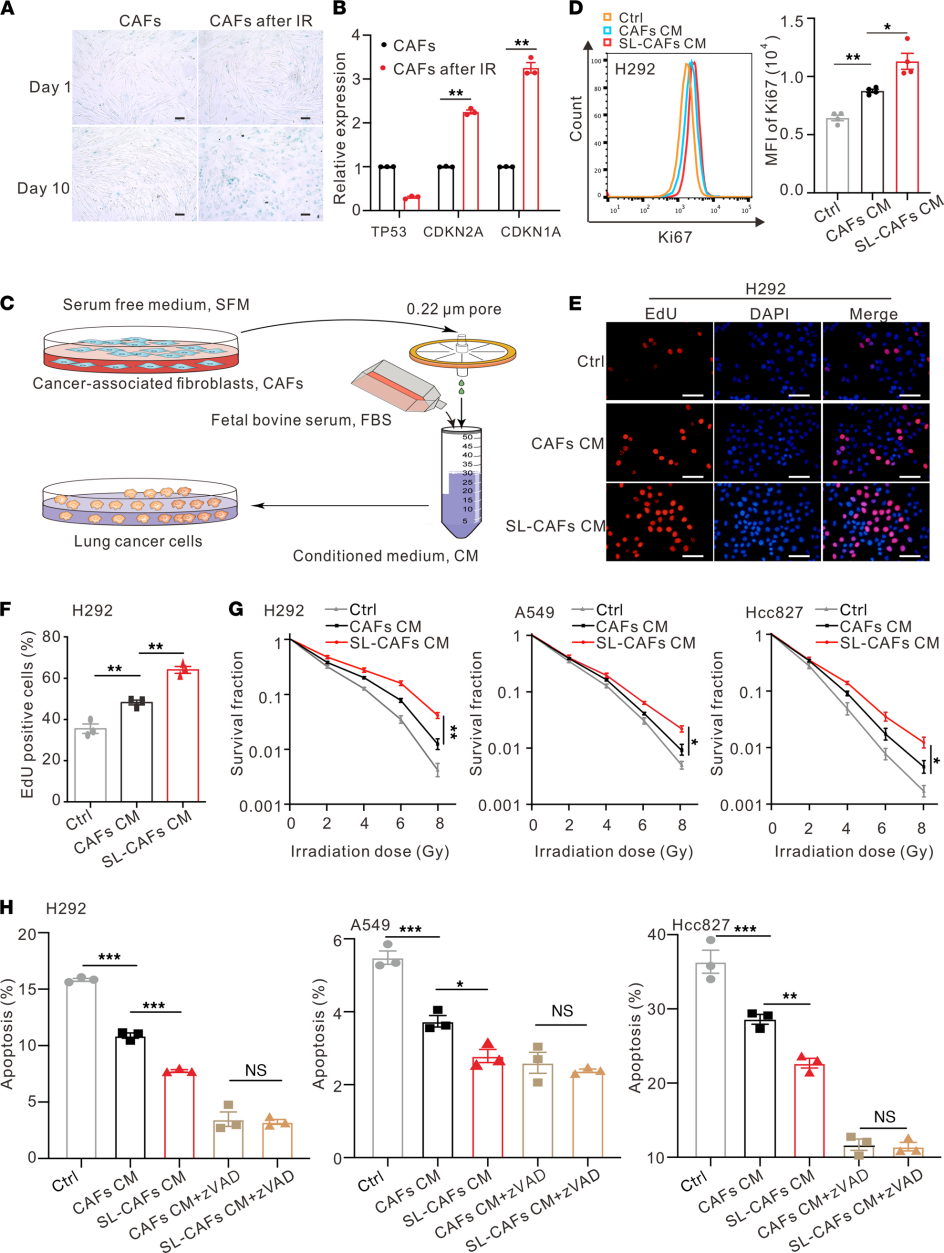
Effects of SL-CAFs on NSCLC cells in vitro. (**A**) Cell senescence detected by SA–β-Gal staining assay. Scale bar: 100 μm. (**B**) Relative mRNA expression of senescence-associated genes in CAFs 10 days after IR. The indicated results represent the mean ± SEM of 4 independent experiments, analyzed by Student’s *t* test. (**C**) Schematic diagram of the CM preparation process. (**D**) Left panel, Ki67 expression of H292 cells cultured with normal medium, CAF CM, or SL-CAF CM for 48 hours detected by flow cytometry. Right panel, statistical analysis of Ki67 expression. The indicated results represent the mean ± SEM of 3 independent experiments, analyzed by 1-way ANOVA. (**E**) Representative images of EdU staining of H292 cells cultured with CAF CM, SL-CAF CM, or control medium for 48 hours. Scale bar: 100 μm. (**F**) Quantification of EdU-positive H292 cells in indicated groups detected by flow cytometry. The indicated results represent the mean ± SEM of 3 independent experiments, analyzed by 1-way ANOVA. (**G**) Clonogenic survival curves of NSCLC cells pretreated with CAF CM, SL-CAF CM, or control medium for 48 hours and then irradiated with 2, 4, 6 or 8 Gy. Cell survival fractions were normalized to those of the unirradiated control cells. The indicated results represent the mean ± SEM of 3 independent experiments, analyzed by 2-way ANOVA. (**H**) NSCLC cells pretreated with CAF CM, SL-CAF CM, or control medium for 48 hours and then irradiated with 8 Gy. The apoptosis rates were measured 72 hours after 8 Gy IR detected by flow cytometry, as indicated in different groups. The indicated results represent the mean ± SEM of 3 independent experiments, analyzed by 1-way ANOVA. *P < 0.05; **P < 0.01; ***P < 0.001; n.s., not statistically significant. SL-CAFs, senescence-like CAFs; zVAD, Z-VAD-FMK.

To study the changes of fibroblasts in the TME after RT, we initially isolated CAFs (Supplemental Figure 1; supplemental material available online with this article; https://doi.org/10.1172/jci.insight.146334DS1) from lung cancer specimens obtained by surgical resections and subjected these to a single 10 gray (Gy) irradiation (IR) treatment. Cell-counting assays demonstrated that the growth of CAFs slowed down after IR and cell growth arrested on the eighth day (Supplemental Figure 2A). In order to confirm the senescent characteristics of CAFs after IR, we tested them for the mRNA expression levels indicative of SASP and found a significant elevation (Supplemental Figure 2B). Senescence-associated β-galactosidase (SA-β-Gal) staining assay showed the positive rate of CAFs increased 10 days after a single IR exposure (Figure 1A and Supplemental Figure 2C). Significantly increased mRNA expression levels of senescence-associated cyclin CDKN1A and CDKN2A genes (12, 25) were detected 10 days after IR, as determined by quantitative PCR (qPCR) analysis (Figure 1B).

The changes of protein expression levels demonstrated by Western blot were consistent with that of mRNA (Supplemental Figure 2D). These results suggest that CAFs have developed as senescence-like CAFs 10 days after IR. Because the tumor-promoting effect of CAFs is usually caused via the secretion of various cytokines, we directly captured the senescent secretome and assessed its function. We collected conditioned media of senescence-like CAFs (senescence-like CAF CM) to culture NSCLC cell lines H292, A549, and Hcc827 for 48 hours, in order to evaluate the influence of senescence-like CAFs on NSCLC cells (Figure 1C). Increased Ki67 expression was detected by flow cytometry in NSCLC cells cultured with senescence-like CAF CM, compared with that of normally growing CAF CM or a control group (Figure 1D and Supplemental Figure 3A). 5-Ethynyl-2’-deoxyuridine (EdU) assays also demonstrated that the senescence-like CAF CM group had more nascent DNA labeled by EdU than the other 2 groups (Figure 1, E and F, and Supplemental Figure 3B). These results reveal that NSCLC cells cultured with senescence-like CAF CM acquire much stronger proliferative ability compared with that of CAF CM. To further explore the effect of senescence-like CAFs on NSCLC cells’ radiosensitivity, NSCLC cells were exposed to single doses of 0, 2, 4, 6, or 8 Gy IR, and their survival was determined by plate clone formation assay. We observed that cancer cells cultured with senescence-like CAF CM had greater radioresistance ability compared with those cultured with CAF CM (Figure 1G). Cell Counting Kit-8 (CCK8) assays were used to detect the effects of CAF CM and senescence-like CAF CM on the cell viability of NSCLC cells 72 hours after 8 Gy IR (Supplemental Figure 4). The results show that the viability of NSCLC cells cultured with senescence-like CAF CM was higher than in those cultured with CAF CM. In order to investigate whether apoptosis played an important role in senescence-like CAF–induced radioresistance of NSCLC cells, annexin V staining analysis was performed to reveal the changes of cell apoptosis rates after a single dose of 8 Gy IR. We found that senescence-like CAF CM reduced IR-induced NSCLC cell apoptosis compared with the CAF CM group, and the difference between these 2 groups could be blocked by the pan-caspase inhibitor Z-VAD-FMK (Figure 1H and Supplemental Figure 5). CCK8 detection also demonstrated that Z-VAD-FMK recovered the difference of growth arrest between the CAF CM group and the senescence-like CAF CM group after IR (Supplemental Figure 4). Taken together, these findings suggest that, compared with CAFs, senescence-like CAFs increase NSCLC cell growth and radioresistance in vitro.

### Senescence-like CAFs induce radioresistance of NSCLC cells via JAK/STAT pathway.

**Figure 2:**
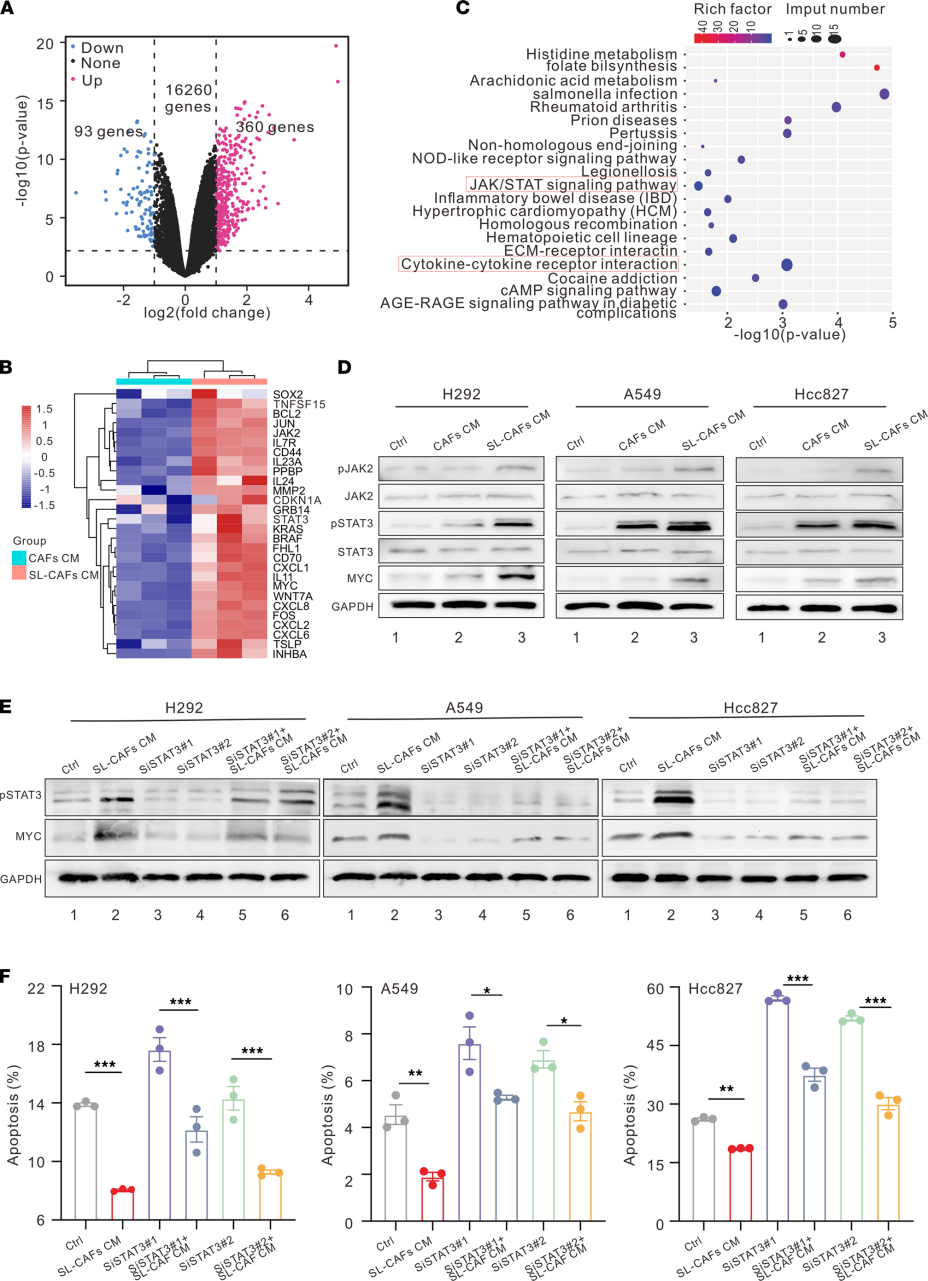
Mechanism of NSCLC cell radioresistance induced by SL-CAFs. (**A**) Volcano plot showing the upregulated, downregulated, or insignificantly differential expressed genes of H292 cells cultured with CAF CM or SL-CAF CM for 48 hours. (**B**) Heatmap illustrating changes of tumor growth– and progression-related gene sets. (**C**) KEGG pathway enrichment revealing the activation of JAK/STAT and cytokine-cytokine receptor interaction signaling pathways as indicated in H292 cells cultured with SL-CAFs. (**D**) Representative Western blot for protein expression levels of pSTAT3, STAT3, pJAK2, JAK2, and MYC in NSCLC cells. (**E**) STAT3 knockdown in NSCLC cells with siRNA and detection of downstream pathway MYC molecules by Western blot. (**F**) NSCLC cell apoptosis rate 72 hours after 8 Gy IR detected by flow cytometry as indicated in different groups. The indicated results represent the mean ± SEM of 3 independent experiments, analyzed by 1-way ANOVA. *P < 0.05; **P < 0.01; ***P < 0.001. SL-CAFs, senescence-like CAFs.

To assess the potential mechanism of radioresistance of NSCLC cells induced by senescence-like CAFs, subsequent RNA-Seq was performed by testing H292 cells cultured with CAF CM or senescence-like CAF CM for 48 hours. Senescence-like CAF CM–treated H292 cells expressed significantly higher levels of tumorigenesis- and progression-related transcription factors, cytokines, and other molecules, such as SOX2, FOS, MYC, CD44, WNT7A, CXCL1, CXCL6, CXCL8, MMP2, BRAF, and KRAS, compared with cells treated in CAF CM (Figure 2, A and B). 

To determine the activated signaling pathway in the senescence-like CAF CM group, Kyoto Encyclopedia of Genes and Genomes (KEGG) analyses based on the differentially expressed genes were performed. JAK/STAT and cytokine-cytokine receptor interaction signaling pathways were enriched in H292 cells cultured with senescence-like CAF CM as determined by the results of KEGG analysis (Figure 2C). As the IL-6/JAK/STAT3 signaling pathway is well established to play an important role in a variety of malignancies by promoting proliferation and inhibiting apoptosis of cancer cells (18, 26–29), Western blot was performed to examine whether the related key markers in this signaling pathway changed in H292, A549, and Hcc827 cell lines cultured with different CM. As shown in Figure 2D, the protein expression levels of phosphorylated STAT3 (pSTAT3) and its downstream target MYC in the senescence-like CAF CM group were upregulated compared with those in the CAF CM group, whereas the control group (NSCLC cells cultured with normal medium) had the lowest protein expression levels. To further demonstrate whether the increase of cell radioresistance was driven by STAT3 in the senescence-like CAF CM group, the STAT3 inhibitor S3I-201 was used to inhibit STAT3 activation. An annexin V staining analysis was performed to reveal the cell apoptosis rates with a single dose of 8 Gy IR. As shown in Supplemental Figure 6, A–C, we discovered that senescence-like CAF CM could reduce IR-induced apoptosis of cancer cells compared with the IR control group, and inhibition of STAT3 could reverse this phenomenon. The clone formation assay corroborated the findings of the cell apoptosis assay, which also demonstrated that S3I-201 (STAT3 inhibitor) could rescue the radioresistance of cancer cells cultured with senescence-like CAF CM (Supplemental Figure 6D). Besides, we also designed 3 different siRNA to knock down STAT3 (Supplemental Figure 7 and Supplemental Table 4) and chose 2 of them for the following experiments. Corroborating the apoptosis results of the STAT3 inhibitor S3I-201 assay, the senescence-like CAF CM reduced IR-induced apoptosis of cancer cells, and knockdown of STAT3 reversed this phenomenon (Figure 2, E and F). Collectively, these findings indicate that senescence-like CAFs induce NSCLC cells’ radioresistance through the JAK/STAT pathway.

### FOXO4-DRI can selectively target senescence-like CAFs and counter NSCLC cells’ radioresistance in vitro.

**Figure 3:**
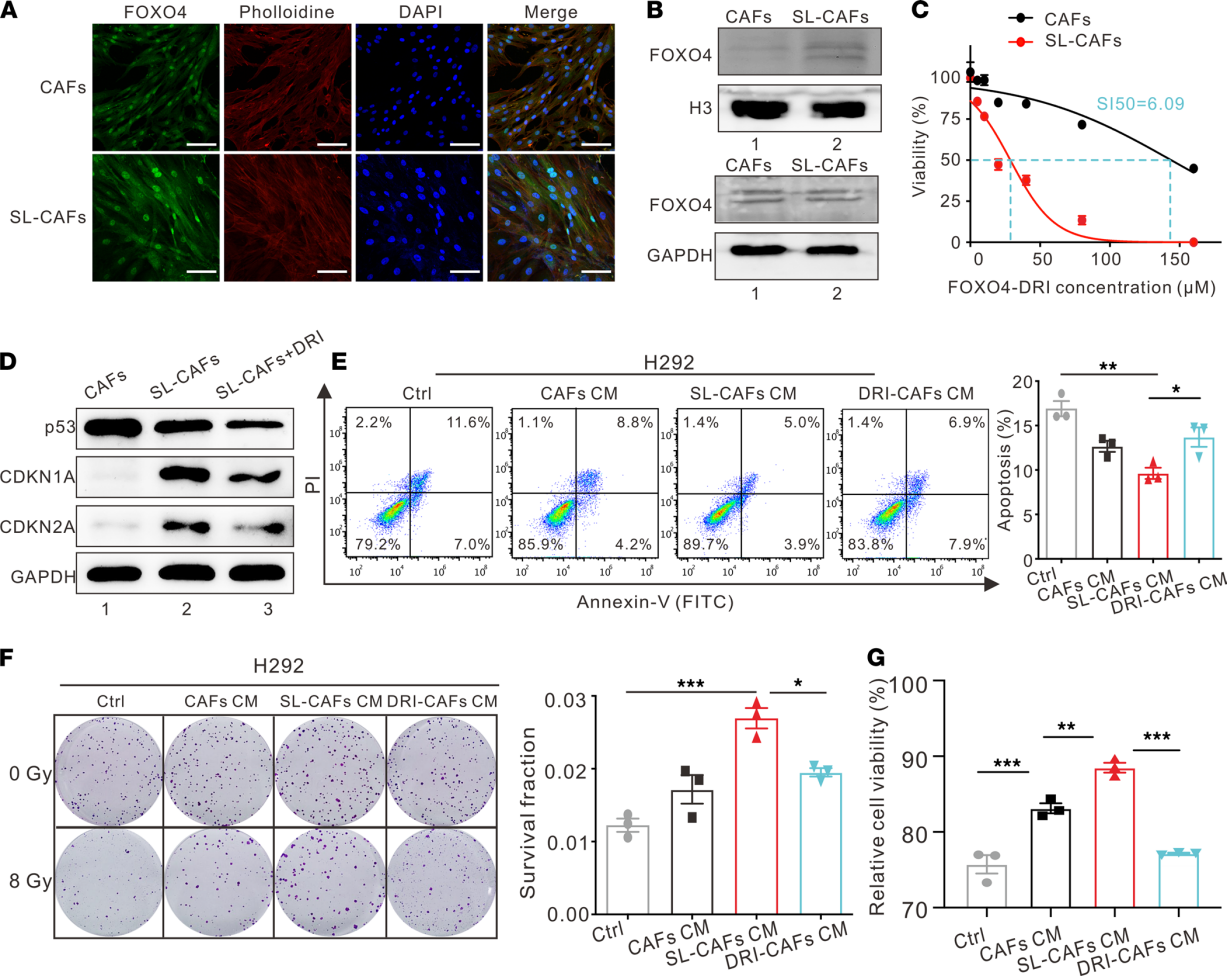
FOXO4-DRI reverses NSCLC cells radioresistance by targeting SL-CAFs. (**A**) Representative images of FOXO4 expression in CAFs and SL-CAFs detected by immunofluorescence staining. Scale bar: 100 μm. (**B**) Western blot for nuclear and total protein expression levels of FOXO4 in CAFs and SL-CAFs. (**C**) Cell viability assay of CAFs and SL-CAFs treated with different concentrations of FOXO4-DRI for 72 hours. The selectivity index (SI50) represents the differences of IC50 between the 2 groups by nonregression analysis. The indicated results represent the mean ± SEM of 3 independent experiments. (**D**) The senescence-associated gene expression changes of SL-CAFs incubated with FOXO4-DRI (50 μM) detected by Western blot. (**E**) H292 cell apoptosis rates after 8 Gy IR detected by flow cytometry and quantitative data of the apoptosis rates as indicated in different groups. The indicated results represent the mean ± SEM of 3 independent experiments, analyzed by 1-way ANOVA. (**F**) Representative images of colony formation in H292 cells cultured in different media after 0 Gy or 8 Gy IR, and statistically clonogenic survival fraction of H292 cells with different media after 8 Gy IR, determined by clone formation. The indicated results represent the mean ± SEM of 3 independent experiments, analyzed by 1-way ANOVA. (**G**) The cell viability of H292 determined 72 hours after 8 Gy IR and normalized to non-IR cells as indicated in different groups. The indicated results represent the mean ± SEM of 3 independent experiments. *P < 0.05; **P < 0.01; ***P < 0.001. SL-CAFs, senescence-like CAFs. DRI-CAFs, senescence-like CAFs incubated with FOXO4-DRI.

FOXO4-DRI was reported to target senescent cell apoptosis effectively by disrupting FOXO4-p53 interaction and causing p53 nuclear exclusion in senescent cells (24). To build on this approach, we assessed whether FOXO4-DRI can be used to target senescence-like CAFs. Because a high expression level of FOXO4 in a senescent cell nucleus is necessary for being selective targeting by FOXO4-DRI, we performed an immunofluorescence experiment to determine the expression difference between senescence-like CAFs and CAFs. As revealed in Figure 3A, the fluorescence intensity of FOXO4 was higher in the nuclei of senescence-like CAFs than in CAFs. The protein expression level of FOXO4 in senescence-like CAF nuclei was higher than that in CAF nuclei, as identified by Western blot assay (Figure 3B). Moreover, FOXO4-DRI selectively reduced the cell viability of senescence-like CAFs and CAFs, with a selective index (SI) of 6.09 (Figure 3C). Also, the protein expression levels of CDKN2A and CDKN1A were markedly elevated in senescence-like CAFs compared with those in CAFs, which can be reversed by incubation with 50 μM FOXO4-DRI (the concentration that effectively targeted senescence-like CAFs but ensured CAF viability more than 80%) for 72 hours, as confirmed by Western blot (Figure 3D). These findings indicate that FOXO4-DRI can clear senescence-like CAFs effectively and selectively in a low concentration.

To further explore whether FOXO4-DRI could decrease NSCLC cells’ radioresistance by clearing senescence-like CAFs, we treated senescence-like CAFs with 50 μM FOXO4-DRI (DRI-CAFs) for 72 hours and then prepared DRI-CAF CM to culture lung cancer cells for 48 hours in the aforementioned manner. Cell apoptosis assays and plate clone formation assays were used to measure cancer cell radiosensitivity after 8 Gy IR. More apoptotic cells were observed in the DRI-CAF CM group than in the senescence-like CAF CM group (Figure 3E and Supplemental Figure 8, A and D). Consistent with the results of cell apoptosis assays, plate clone formation assays also revealed that the DRI-CAF CM group had fewer clones and a lower surviving fraction than the senescence-like CAF CM group (Figure 3F and Supplemental Figure 8, B and E). CCK8 assays were used to detect the NSCLC cells’ viability under 8 Gy IR after FOXO4-DRI treatment (Figure 3G and Supplemental Figure 8, C and F). The results show that the NSCLC cells cultured with DRI-CAF CM were significantly less viable than those in senescence-like CAF CM. These findings indicate that FOXO4-DRI can reduce radioresistance of NSCLC cells induced by senescence-like CAFs in vitro. Collectively, FOXO4-DRI can effectively increase NSCLC cells’ radiosensitivity by targeting senescence-like CAFs.

### FOXO4-DRI can enhance NSCLC cells’ radiosensitivity by targeting senescence-like CAFs in vivo.

**Figure 4:**
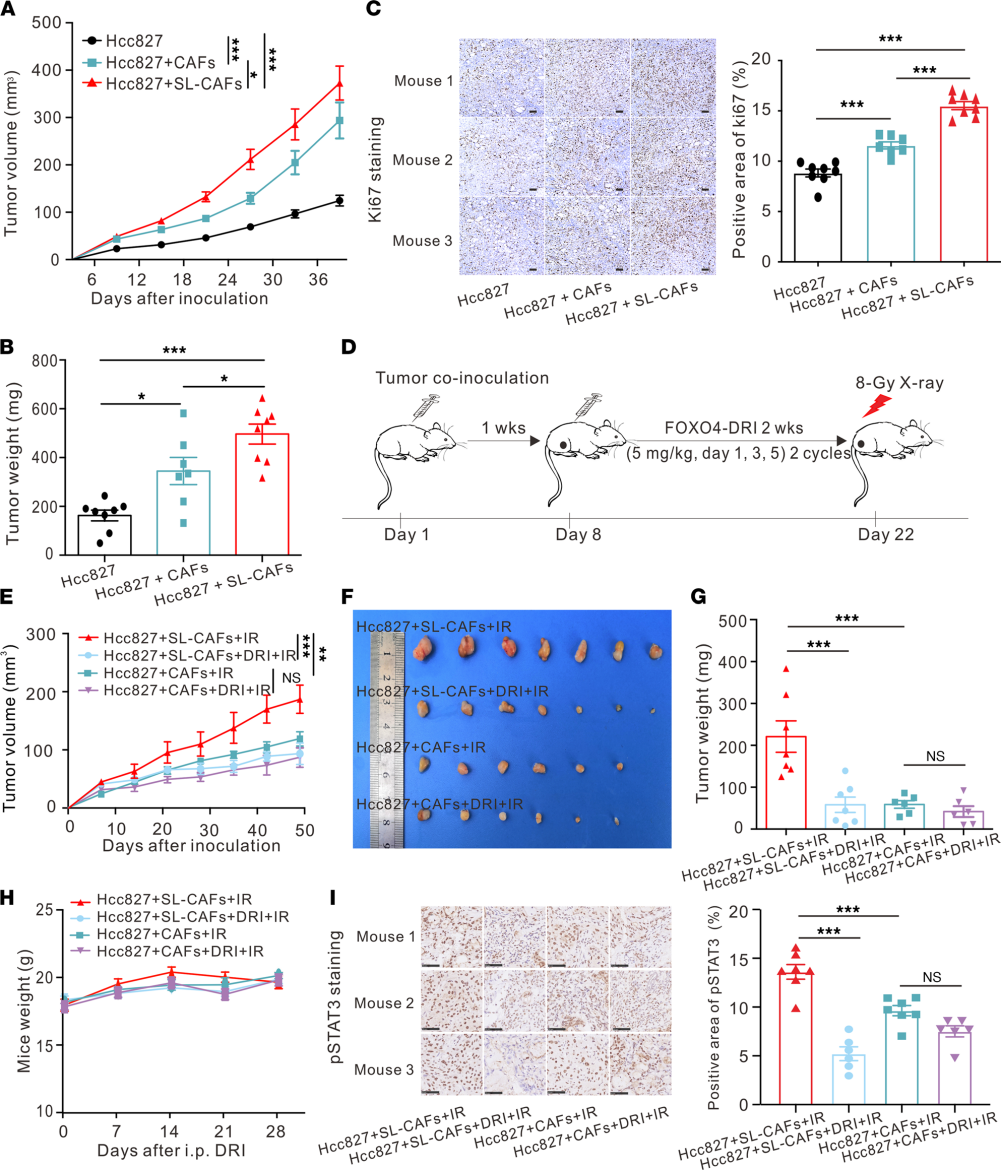
Effect of FOXO4-DRI in improving radiosensitivity in vivo. (**A**) Tumor growth curves for mice s.c. coinjected with Hcc827 cells with SL-CAFs or CAFs. Hcc827 cells injected alone as control. Mean ± SEM (*n* = 7–8 mice per group), analyzed by 2-way ANOVA. (**B**) Tumor weight for mice s.c. coinjected with Hcc827 cells with SL-CAFs or CAFs. Hcc827 cells injected alone as control. Mean ± SEM (*n* = 7–8 mice per group), analyzed by 1-way ANOVA. (**C**) Representative images of the Ki67 immunohistochemistry staining in dissected tumors (Scale bar: 100 μm), and semi-quantitative analysis of positive area of Ki67 immunohistochemistry staining by ImageJ software (NIH). Mean ± SEM (original magnification, 400×; 3 microscopic fields per mouse to evaluate the averaged ki67^+^ area, *n* = 7–8 mice per group), analyzed by 1-way ANOVA. (**D**) Schematic of experiment to assess FOXO4-DRI–improved radiosensitivity In mouse model. (**E**) Tumor growth curves for mice in different groups as indicated. Mean ± SEM (*n* = 6–7 mice per group), analyzed by 2-way ANOVA. (**F**) Photo of dissected tumors and (**G**) quantification of tumor weights in each group. Mean ± SEM (*n* = 6–7 mice per group), analyzed by 1-way ANOVA. (**H**) Mouse weights measured at indicated time points. (**I**) Representative images of the pSTAT3 immunohistochemistry staining in dissected tumors of different groups (Scale bar: 50 μm), and semi-quantitative analysis of positive area of pSTAT3 immunohistochemistry staining by ImageJ software. Mean ± SEM (Original magnification, 400×; 3 microscopic fields per mouse to evaluate the averaged pSTAT3 area; *n* = 6–7 mice per group), analyzed by 1-way ANOVA. *P < 0.05; ***P < 0.001; n.s., not statistically significant. SL-CAFs, senescence-like CAFs.

After we demonstrated that senescence-like CAFs promoted NSCLC cell proliferation in vitro, we examined whether senescence-like CAFs could accomplish the same role in vivo. Senescence-like CAFs or CAFs were s.c. coinoculated with Hcc827 or H292 cells in athymic nude mice. Control-group mice were inoculated with Hcc827 or H292 cells alone. Tumor engraftments were 100% in all groups. Compared with inoculating NSCLC cells alone, coinoculation of CAFs or senescence-like CAFs promoted tumor growth and led to increased tumor volumes and tumor weights (Figure 4, A and B; Supplemental Figure 9A; and Supplemental Figure 10, A–C). Furthermore, IHC staining with Ki67 revealed that coinoculation with senescence-like CAFs had greater positive area than that of CAFs (Figure 4C). Overall, these results indicate that compared with CAFs, senescence-like CAFs significantly enhance lung cancer growth in vivo.

To assess whether FOXO4-DRI can radiosensitize tumor cells in vivo, we combined RT with FOXO4-DRI to treat s.c. xenografts of Hcc827 or H292 cells coinoculated with senescence-like CAFs or CAFs (Figure 4D). Treatment with FOXO4-DRI significantly increased radiosensitivity and decreased tumor volumes and weights in the senescence-like CAFs coinoculation group (Figure 4, E–G, and Supplemental Figure 10, D–F). However, treatment with FOXO4-DRI in the CAFs coinoculation group did not increase radiosensitivity. In mice administered with the FOXO4-DRI group, no significant weight loss was observed (Figure 4H). We also tested the radiosensitization effect of FOXO4-DRI in an immune-competent C57BL/6 mouse model, combining RT with FOXO4-DRI to treat s.c. xenografts of Lewis cells. As shown in Supplemental Figure 11, A–C, the radiosensitization effect of FOXO4-DRI in the combined treatment group was significant. To further elucidate the selective depletion of senescence-like CAFs by FOXO4-DRI, CAFs were transfected with a green fluorescent protein (GFP) tag (CAFs-GFP) and irradiated with 10 Gy to obtain senescence-like CAFs-GFP. Then, the CAFs-GFP or senescence-like CAFs-GFP with Hcc827 cells were s.c. coinoculated in the nude mice. As shown in Supplemental Figure 9C, the GFP^+^ cells in the senescence-like CAFs group were eliminated by FOXO4-DRI, whereas the GFP^+^ cells in the CAFs group could still be found. These findings suggest that FOXO4-DRI can enhance tumor cells’ radiosensitivity by targeting senescence-like CAFs in vivo. Furthermore, we explored the specific mechanisms by which FOXO4-DRI promotes tumor radiosensitivity. As shown in Supplemental Figure 9B, IHC staining of cleaved caspase 3 revealed that FOXO4-DRI enhanced cancer cells’ radiosensitivity by promoting cell apoptosis in vivo. IHC staining of pSTAT3 showed that the growth rate upon tumor inoculation with senescence-like CAFs was highest and decreased significantly with FOXO4-DRI treatment, consistent with the in vitro results that senescence-like CAFs induced radioresistance of cancer cells by activating the pSTAT3 pathway (Figure 4I). In summary, these findings suggest that FOXO4-DRI can reduce the pSTAT3 activity of tumor cells and promote tumor cell apoptosis by targeting senescence-like CAFs in vivo.

### FOXO4-DRI can alleviate RIPF by targeting senescence-like fibroblasts.

**Figure 5:**
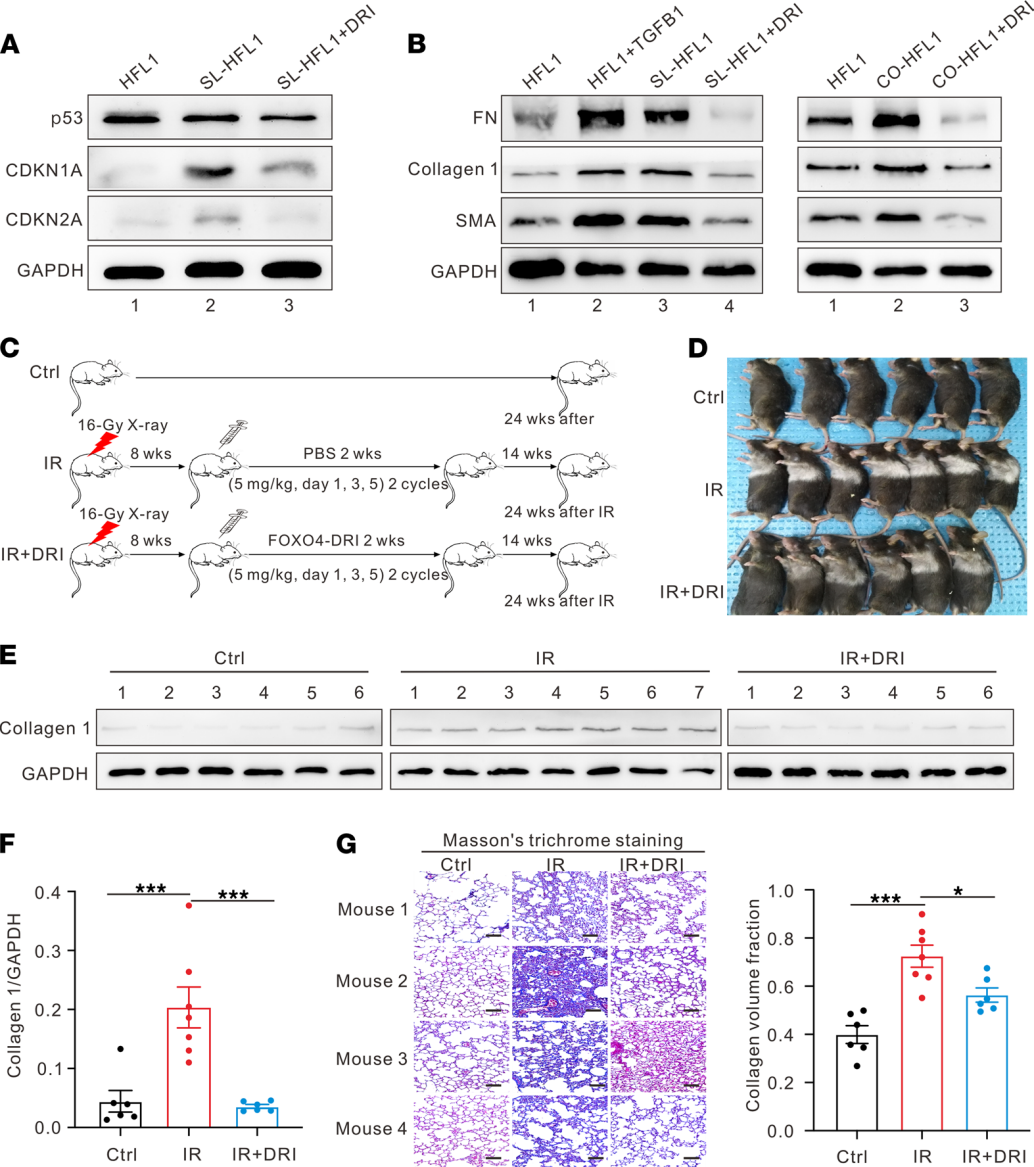
FOXO4-DRI alleviates RIPF. (**A**) Representative Western blot for the senescence-associated gene expression changes of SL-HFL1 and SL-HFL1 incubated with FOXO4-DRI (20 μM). (**B**) Western blot showing analysis demonstrates the fibrotic gene expression changes of SL-HFL1 or CO-HFL1, and SL-HFL1 or CO-HFL1 incubated with FOXO4-DRI. HFL1 incubated with TGF-β1 (20 ng/mL) used as positive control. (**C**) Schematic of animal experiment. (**D**) Photographs of mice in different groups as indicated. (**E**) Western blot analysis examining collagen 1 expression in mouse lung tissues and (**F**) quantification of changes of expression levels based on gray value. Mean ± SEM (*n* = 6–7 mice per group), analyzed by 1-way ANOVA. (**G**) Representative images of histological changes in mouse lungs evaluated by Masson′s trichrome staining (Scale bar: 100 μm), and semi-quantitative image analysis of collagen content (blue areas) in lungs. Mean ± SEM (Original magnification, 400×; 3 microscopic fields per mouse to evaluate the averaged collagen content; *n* = 6–7 mice per group), analyzed by 1-way ANOVA. *P < 0.05; ***P < 0.001. SL-HFL1, senescence-like HFL1. CO-HFL1, HFL1 cocultured with senescence-like HFL1.

With the inevitable exposure of normal lung tissue to radiation, RIPF is one of the most severe complications following thoracic RT. To further examine the role of radiation-induced senescence-like fibroblasts in the development of RIPF, human lung fibroblast cell line HFL1 was irradiated to induce senescence-like HFL1 (SL-HFL1) in the aforementioned manner. The SI of FOXO4-DRI on the cell viability of SL-HFL1 and HFL1 was 3.51 (Supplemental Figure 12). As shown in Figure 5A, the protein expression levels of CDKN2A and CDKN1A were higher in the SL-HFL1 group than in the HFL1 group; incubating SL-HFL1 with 20 μM FOXO4-DRI (DRI-HFL1) could decrease the high protein expression levels of CDKN2A and CDKN1A, as demonstrated by Western blot. Increased protein expression levels of α smooth muscle actin (α-SMA), type 1 collagen (collagen 1), and fibronectin (FN) were found in both SL-HFL1 or HFL1 cocultured with SL-HFL1 for 72 hours (CO-HFL1); however, these expression level increases could be reversed by FOXO4-DRI incubation (Figure 5B). These findings indicate that senescence-like fibroblasts induced by radiation can develop into fibrotic phenotypes like myofibroblasts.

To further explore whether FOXO4-DRI could be used to treat RIPF, a single dose of 16 Gy thoracic radiation was given to C57BL/6 mice to establish the RIPF model. Mice without IR were used as control. Figure 5C illustrates the treatment process. The hair color of the mice with IR alone turned white gradually, whereas the hair color of the mice with IR combined with FOXO4-DRI was gray (Figure 5D). All mice were sacrificed 24 weeks after IR, and their lungs were collected for future experiments. Higher type 1 collagen protein expression levels were found in the IR group compared with the control group without IR, and combined treatment with FOXO4-DRI downregulated the type 1 collagen protein expression level (Figure 5, E and F). Consistent with this result, Masson’s trichrome staining of mouse lung tissues also identified fibrosis after IR, and FOXO4-DRI could significantly reverse RIPF with fewer collagen depositions (Figure 5G). These results suggest that FOXO4-DRI can effectively alleviate RIPF in vivo.

## Discussion

Credible evidence indicates that CAFs are protumorigenic and expected to serve as potential targets for anticancer therapy (7–9). Compared with strategies to block CAFs’ paracrine signaling pathways or deplete CAF-derived ECM proteins related to tumor cells’ negative biological behaviors, fundamentally clearing CAFs is likely to be more direct and efficient. In recent years, the heterogeneous characteristics of CAFs in the TME have been demonstrated, and their diverse subsets have been defined according to various different phenotypes or functions (30–33). However, strategies of targeting CAF subsets via specific biomarkers may potentially result in multiple adverse outcomes, even leading to worse survival, which hinders their application in cancer therapy (34, 35). In this scenario, there is an urgent need to explore effective targets for clearing CAFs in order to achieve improved antitumor efficacy. Here, we reported that radiation-induced senescence-like CAFs had a prominent ability to increase NSCLC cells’ radioresistance. Consistent with our studies, Tsai et al. have also reported that low-dose radiation-induced senescent mammary fibroblasts can render nearby breast cancer cells radioresistant (36). In contrast with their study, we focused on targeting senescence-like CAFs rather than CAFs; consequently, we present a feasible strategy to clear senescence-like CAFs that can significantly sensitize radiosensitivity of NSCLC cells both in vitro and in vivo. Compared with other strategies of targeting CAFs via specific biomarkers, the functionally targeted clearance of senescence-like CAFs not only avoids the limitation of low-clearance efficiency caused by CAF heterogeneity, but it also solves the dilemma of excessive clearance of normal fibroblasts caused by cross-markers on the cell membrane. Thus, strategies based on the clearance of senescence-like CAFs could be a more rational and effective approach in anticancer therapy. In our study, we chose Hcc827 and H292 (TP53 mutant and WT) cells for the in vivo experiments, and the FOXO4-DRI were used to target senescence-like CAFs in order to reduce NSCLC cells’ radioresistance. However, whether the FOXO4-DRI has a direct killing effect on senescent tumor cells needs further evaluation. For the in vitro experiments, though RNA-Seq and Western blot indicate that pSTAT3 and MYC may play important roles in senescence-like CAF–induced radioresistance in NSCLC cells, whether there are other pathways involved in the radiosensitivity needs further investigation. For example, given that SASP factors influence the progression, stemness, and migration of malignant cancer (11–13), it remains an interesting question whether chronic SASP exposure can affect cancer cells’ radiosensitivity due to stem-like pathways changes.

Currently, more attention has been paid to the role of senescence-like fibroblasts rather than fibroblasts in affecting cancer cells. Previous studies have shown that a single dose of 10 Gy radiation can induce senescent phenotypes in fibroblasts because of the consequential DNA damage without causing cell death. Abundant evidence has shown that radiation-induced senescence-like fibroblasts are characterized by mass secretions of SASP, especially IL-6 and IL-8, exercising protumorigenic functions by reducing cell apoptosis or promoting cell survival (37). We detected tumor cell apoptosis rates 72 hours after IR and found that apoptosis may play an important role in senescence-like CAF–mediated radioresistance. However, whether there are other events of cell death involved requires more in-depth research. In addition, our study showed that senescence-like CAFs induced by a 10 Gy radiation dose acquired a much stronger ability to promote NSCLC growth than CAFs do in vivo. Other research groups have used long-passaged fibroblast cell lines or normal tissue–derived primary fibroblasts, which might lose characteristics of CAFs or possess characteristics different from CAFs. In contrast, we isolated primary CAFs from lung cancer tissues donated by multiple patients. However, the overall role of radiation-induced senescence-like CAFs on regulating tumor growth may be debated. Hellevik et al. reported that after exposure to a single 18 Gy radiation dose, CAFs isolated from NSCLC patient tissues developed senescent phenotypes without significant secretion changes of IL-6 and IL-8, which is obviously distinct from secretion profiles of 10 Gy radiation–induced CAFs (38, 39). Grinde et al. reported that a single high dose (1 × 18 Gy) or fractionated dose (3 × 6 Gy) abrogated the protumorigenic ability of CAFs coinjected in murine lung cancer xenografts (40). Possible interpretations may lie in the different RT doses and fraction regimens used, which lead to changes in the CAFs’ secretomes and influence their crosstalk with cancer cells (41). Hence, the profound effects of RT on CAFs and the crosstalks between CAFs and cancer cells require further studies.

When it comes to thoracic RT, RIPF is one of the most severe complications in patients who receive definitive thoracic RT because of the inevitable exposure of normal lung tissues to radiation. RIPF is characterized by irreversible structural destruction of lung tissues and deterioration of lung function, eventually leading to respiratory failure and death (42). Potential mechanisms under RIPF include the release of reactive oxygen species (ROS) and inflammatory cytokines, activation of TGF-β signaling pathways and development of the epithelia-mesenchymal transformation (EMT), abnormal repair of cellular injuries, and excessive ECM deposition (43). Unfortunately, no effective medical therapy targeting these causes has been approved for preventing or reversing RIPF progression. The only 2 drugs (pirfenidone and nintedanib) approved for clinical use to treat idiopathic pulmonary fibrosis (IPF), have not demonstrated effectiveness for treating RIPF. Accumulating evidence indicates that cell senescence may be an important pathogenic factor in PF, including RIPF, bleomycin-induced PF, and IPF (23). Strategies to eliminate senescent cells have been proven to restore pulmonary compliance, structure, and elasticity, subsequently improving pulmonary function in aging mice (21, 44). As one of the senolytic drugs targeting senescent cells, ABT-263 (Bcl-2 family inhibitor) has been reported to reverse RIPF by selectively inducing apoptosis of radiation-induced senescent type II alveolar epithelial cells (AECII) in mice (45). However, the strategy of targeting senescence-like fibroblasts for RIPF treatment had not been proposed before, to the best of our knowledge. Given that fibroblasts may be transformed into senescence-like fibroblasts after radiation, we speculated that senescence-like fibroblasts might contribute to the pathogenesis of RIPF as senescence-like fibroblasts in PF and bleomycin-induced IPF (46, 47). In this study, we find that senescence-like fibroblasts induced by radiation possess the characteristics of myofibroblasts with an increasing expression of fibrotic markers in vitro. It is worth noting that myofibroblasts have been reported to play a crucial role in the pathogenesis of PF via synthesizing and regulating ECM deposition. Thus, we used FOXO4-DRI to target the senescent fibroblasts selectively and observed the remarkable effect on decreasing fibrotic phenotypes in vitro and alleviating collagen deposition in the lung tissues of mice receiving thoracic radiation. Compared with ABT-263, FOXO4-DRI has a better selective target effect on senescent fibroblasts, yet it is safer to normal cells in vitro, as reported by Baar et al. (24). In vivo comparative studies of these 2 agents are necessary; in addition, the mechanism of senescence-like fibroblasts in the development of RIPF also requires investigation in the future. Sex is one of the biological variants for radiation-induced lung injury (48, 49). The incidence and prognosis differences between men and women are also related to their smoking history, RT mode, and chemotherapy regimen (50–52). In our study, we used male mice for chest RT because the body size of male mice is generally larger than that of females. Chest RT could be implemented better with a larger chest area to avoid radiation damage to surrounding tissues caused by respiratory movement or setup errors. Whether FOXO4-DRI has a curative effect for women remains to be investigated. RT can lead to oxidative stress–induced senescence (53). Oxidative stress can contribute to hair follicle damage and bulbar melanocyte migration, malfunction, and death (54, 55). We speculate that oxidative stress caused by RT leads to mice developing white hair, as in Figure 5D. However, the specific mechanisms of senescence caused by oxidative stress and whether FOXO4-DRI can reverse senescence through this pathway remain to be further explored.

## Methods

### Primary and cell line cultures.

All human primary CAFs were isolated from fresh lung cancer specimens obtained by surgical resections in Union Hospital, Tongji Medical College, Huazhong University of Science and Technology, Wuhan, China. Pathology and demographic information of the patients whose clinical samples were used for isolation of CAFs is shown in Supplemental Table 1. Primary CAFs were isolated and cultured as follows. Tumor tissues were cut into 1–2 mm^3^ pieces and washed in PBS 3 times. Subsequently, the tissue pieces were attached to the wall of T25 cell culture flasks and cultured in 1 mL DMEM medium (Thermo Fisher Scientific) supplemented with 10% FBS (Thermo Fisher Scientific) and 1% penicillin/streptomycin. The cell medium was changed every other day, and the tissue pieces were always kept adherent to the flasks. CAFs could be harvested after an approximately 2-week culture as more and more fibroblasts swam out. The purity of CAFs was validated by immunofluorescent staining or flow cytometry, which showed that CAFs were negative for epithelial cell adhesion molecule (EpCAM), CD45, and CD31, and positive for α-SMA and fibroblast activation protein (FAP; > 95%). CAFs between 2 and 6 passages were used for experiments. All cell lines were purchased from American Tissue Culture Collection (ATCC). The human NSCLC cell lines, NCI-H292, A549, and Hcc827, were cultured in RPMI 1640 medium (Thermo Fisher Scientific) with 10% FBS and 1% penicillin/streptomycin. The murine Lewis lung cancer cell line was cultured in DMEM medium (Thermo Fisher Scientific) with 10% FBS and 1% penicillin/streptomycin. The human fetal fibroblast cell line HFL1 was cultured in Ham’s F12K medium (Thermo Fisher Scientific) with 10% FBS and 1% penicillin/streptomycin. All cells were cultured at 37°C in a humidified atmosphere of 5% CO_2_. Routine tests for cell mycoplasma infection were perform and confirmed to be negative.

### Radiation.

CAFs cultured in 10 cm dishes were irradiated by single doses of 2, 4, 6, 8, and 10 Gy. For the s.c. tumor IR, mice were anesthetized; then, the right posterior limbs (with tumors) were exposed to a single dose of 8 Gy radiation. Standard parameters for dose delivery were beam quality of 6 MV and dose rate of 6 Gy/min. Radiation doses were confirmed by thermo-luminescent dosimeters (TLDs).

### Cell growth curve.

CAFs were seeded in 12-well plates (5000 cells per well) and allowed to grow for 24 hours before IR. Cells were harvested and then counted with a Countstar Automated Cell Counter (IC1000) on days 0, 2, 4, 6, 8, and 10 after IR.

### SA–β-Gal staining assay.

The indicated cells were washed with PBS and fixed with 4% paraformaldehyde (Servicebio) for 15 minutes. Then, the cells were stained with a Senescence Cells Histochemical Staining Kit (CS0030-1KT, MilliporeSigma), according to manufacturer instructions.

### Conditioned medium (CM).

To generate CM, fibroblasts were firstly seeded in 10 cm cell dishes and cultured in DMEM supplement with 10% FBS and 1% penicillin/streptomycin. When the cell density reached 90%, the medium was replaced by 5 mL of serum-free 1640 medium with 1% penicillin/streptomycin. Then the serum-free medium was collected after 6 hours of culture and filtered with a 0.22 μm pore (MilliporeSigma). A total of 10% FBS was added to generate CM when used for NSCLC cell cultures.

### Cell viability assay.

To measure the selective killing effect of FOXO4-DRI, indicated cells were plated in 96-well plates (8000 senescent cells and 2000 nonsenescent cells per well). The culture medium was replaced by 200 μL of fresh medium containing different concentrations of FOXO4-DRI (0, 5, 10, 20, 40, 80, and 160 μM). After 72 hours of incubation, cell viability was determined by a CCK-8 assay kit (BS350B, Biosharp).

### qPCR.

RNA was extracted from CAFs and senescence-like CAFs using a MicroElute Total RNA Kit (Omega Bio-tek). At that time, the reverse transcription assay was conducted with HiScript III RT SuperMix (+gDNA wiper) (Vazyme). qPCR was performed on a StepOnePlus Real-Time PCR System (Thermo Fisher Scientific) using AceQ Universal SYBR qPCR Master Mix (Vazyme) to amplify cDNA for 40 cycles. GAPDH expression was used for normalization. The sequence of each primer is listed in Supplemental Table 3. All primers in the study were synthesized by Sangon Biotech.

### Immunofluorescence staining.

For cell immunofluorescence staining, the indicated cells were seeded on 20 mm round coverslips and allowed to adhere. Subsequently, cells were fixed with 4% paraformaldehyde for 15 minutes and permeabilized with 0.5% Triton X-100 (Servicebio) for 5 minutes, followed by washing with PBS 3 times. After blocking with 5% BSA, cells were incubated with anti-FOXO4 antibody (ab63254, Abcam, 1:200 diluted in PBS) overnight at 4°C. Subsequently, cells were stained with the FITC conjugated goat anti–rabbit IgG (GB22303, Servicebio, 1:200) for 1 hour. Cell structures were stained using Pholloidine (G1041, Servicebio, 1:200), and nuclei were dyed with DAPI (G1012, Servicebio, 1:200) for 10 minutes at room temperature. After washing with PBS, cell immunofluorescence was visualized by confocal fluorescence microscope (Leica TCS SP8).

### IHC staining and analysis.

Tumor tissues were sent to Biossci Biotechnology Co. Ltd. for paraffin embedding and serial sectioning after fixation. After a serial process of deparaffinization, antigen unmasking, and blocking, slides were incubated with a mouse anti-Ki67 antibody (ab16667, Abcam, 1:5000) overnight at 4°C. Thereafter, the slides were stained with DAB and counterstained with hematoxylin. Image-pro Plus 6.0 software (Media Cybernetics Inc.) was used to select the same brown as the unified standard for judging the positivity of all photos and to analyze each photo to get the positive area.

### Masson’s trichrome staining.

Lung tissues were sent to Biossci Biotechnology Co. Ltd. for paraffin-embedding and serial sectioning after fixation. Masson’s Trichrome Staining Solution (BA4079B, Baso) was used to perform Masson’s trichrome staining according to the manufacturer’s protocol.

### Clonogenic assay.

NSCLC cells were pretreated with indicated CM for 48 hours. Appropriate numbers of cells were seeded into 6-well plates depending on the different radiation doses and exposed to 0, 2, 4, 6, and 8 Gy radiation. The plating efficiencies (PE) in different groups are shown in Supplemental Table 2. Cells were further allowed to grow for 10–14 days to form clusters, followed by 4% paraformaldehyde fixation and crystal violet (G1014, Servicebio) staining. Colonies comprising more than 50 cells were counted. The calculation formulae for PE and survival fraction (SF) are as follows: PE = number of colonies counted/number of colonies seeded. SF = (number of colonies counted/number of colonies seeded)_test_/(number of colonies counted/number of colonies seeded)_control_, where “test” denotes the test condition (some radiation dose) and “control” denotes identical cells without radiation.

### Apoptosis assay.

Before exposure to 8 Gy radiation, NSCLC cells were seeded into 6-well (2 × 10^5^ per well) plates and pretreated with indicated CM for 48 hours. Seventy-two hours after IR, cells were washed with PBS and harvested by trypsin without EDTA. Then cells were labeled with an Annexin V-FITC Cell Apoptosis Detection Kit (Biobox) and analyzed by flow cytometry.

### Western blot.

Protein samples from cells were prepared using RIPA lysis buffer (50 mM Tris [pH 7.4], 150 mM NaCl, 1% NP-40, 0.5% sodium deoxycholate) and resolved by SDS-PAGE; they were then transferred to polyvinylidene fluoride (PVDF) membranes. Western blots were performed under standard conditions using the indicated antibodies. After incubation with the appropriate secondary antibodies conjugated to horseradish peroxidase (HRP), the quantification of protein expression levels was revealed using an ultra-sensitive ECL luminescence reagent (MA0186, Dalian Meilun Biotechnology Co. Ltd.) with an infrared imaging system (UPV ChemiDoc-It 510, Thermo Fisher Scientific).

### Animal experiment.

Female BALB/c nude mice (5 weeks old) and male C57BL/6 mice (6 weeks old) were purchased from Hubei provincial center for disease control and prevention (HBCDC). For the animal experiment to verify the tumor-promoting effect of senescence-like CAFs in vivo, 3 × 10^6^ Hcc827 or H292 cells with or without indicated fibroblasts were s.c. coinjected at the right posterior limb of nude mice. Tumor sizes were measured every 6 days using vernier calipers. For the radiosensitization effect of FOXO4-DRI in vivo, Hcc827 or H292 cells and indicated fibroblasts (ratio 1:2) were s.c. coinjected at the right posterior limb of nude mice. After 7 days, mice were randomly divided into different groups and treated with i.p. injections of PBS or FOXO4-DRI (5 mg/kg per mouse on days 1, 3, and 5 every week for 2 cycles). Tumor sizes were measured every week using vernier calipers. Tumor volume (*V*) was calculated by the formula *V*= (Length × Width^2^)/2. When average tumor volume reached about 50–80 mm^3^, a single dose of 8 Gy x-ray was given to treat the tumor. After mice were euthanized, the tumors were photographed and weighed. In the PF model, C57BL/6 mice received a single 16 Gy thoracic radiation. After 8 weeks, PBS or FOXO4-DRI (5 mg/kg per mouse on days 1, 3, and 5 every week for 2 cycles) were i.p. injected into mice. After 24 weeks, mice were sacrificed, and their lungs were collected for future experiments.

### EdU proliferation assay.

H292, A549, and Hcc827 cells were seeded in 96-well plates (5 × 10^3^ cells per well) cultured with normal medium, CAF CM, or senescence-like CAF CM for 48 hours, respectively. Cell proliferation was determined by the EdU Apollo in Vitro Imaging Kit (RiboBio) according to the manufacturer’s protocol, and cells were exposed to EdU before staining for 2 hours.

### RNA-Seq analysis.

RNA-Seq was performed by Seqhealth Technology Co. Ltd. Raw sequencing data were filtered by Trimmomatic (version 0.36) to obtain clean data, which were then mapped using STRA software (version 2.5.3a). Reads mapped to the exon region of each gene were counted by using featureCounts (Subread-1.5.1; Bioconductor), and then RPKMs were calculated. Differential gene expression between 2 groups was identified by using the edgeR package (version 3.12.1). A fold-change cutoff of 2 and *P* value cutoff of 0.05 were used to judge the statistical significance of gene expression differences. KEGG enrichment analysis was implemented by KOBAS software (version: 2.1.1) to judge statistically significant enrichment. We have uploaded the RNA-Seq data to the GEO public database (GSE185698).

### Statistics.

All the data were presented as the mean ± SEM from at least 3 independent experiments. For comparisons of 2 groups, a 2-tailed unpaired Student’s *t* test was performed. For comparisons of 3 or more groups, 1-way ANOVA was performed with Tukey’s multiple-comparison test. One-way ANOVA was also used for comparing 2 groups in multiple-group comparisons. Tumor growth was assessed by 2-way ANOVA. Statistical analysis was performed with GraphPad Prism 6.0 software (GraphPad Software Inc.). *P* < 0.05 was considered to be statistically significant. Data are presented as mean ± SEM. *P < 0.05; **P < 0.01; ***P < 0.001.

### Study approval.

All animal experiments were approved by the IACUC at Tongji Medical College, Huazhong University of Science and Technology. Human studies were approved by the Medical Ethics Committee at Union Hospital, Tongji Medical College, Huazhong University of Science and Technology. Written informed consent was obtained from the participants prior to the study.

## Author contributions

JM, YL, and CW performed the experiments and wrote the draft. YS, XD, and JH analyzed the data and helped write the draft. Yan Hu, YG, BW, and ZZ developed the methodology. KJ, SX, JFL, Yu Hu, and GW reviewed and revised the manuscript. KY and HJ conceived the project and revised the manuscript.

## Supplementary Material

Supplemental data

## Version 1 12/08/2021

Electronic publication
