# Supplementary material for: Targeting senescence-like fibroblasts radiosensitizes non–small cell lung cancer and reduces radiation-induced pulmonary fibrosis
Source: JCI Insight. 2021 Dec 8;6(23):e146334. doi: 10.1172/jci.insight.146334 (PMC8675198; doi:10.1172/jci.insight.146334)
Supplement: Supplemental data [file jciinsight-6-146334-s076.pdf]

Targeting senescence-like fibroblasts radiosensitizes non-small cell lung cancer and reduces radiation-induced pulmonary fibrosis

Jingshu Meng[†1], *Yan Li*[†1], *Chao Wan*[†1], *Yajie Sun*[†], *Xiaomeng Dai*[†], *Jing Huang*[†], *Yan Hu*[†], *Yanan Gao*[†], *Bian Wu*[†], *Zhanjie Zhang*[†], *Ke Jiang*[#], *Shuangbing Xu*[†], *Jonathan F. Lovell*[‡], *Yu Hu*[^], *Gang Wu*[†], *Honglin Jin*[†*], *and Kunyu Yang*[†*]

[†]Cancer Center, Union Hospital, Tongji Medical College, Huazhong University of Science and Technology, Wuhan 430022, China

[#]Department of Thoracic Surgery, Union Hospital, Tongji Medical College, Huazhong University of Science and Technology, Wuhan 430022, China

[^]Institute of Hematology, Union Hospital, Tongji Medical College, Huazhong University of Science and Technology, Wuhan 430022, China

[‡]Department of Chemical and Biological Engineering, University at Buffalo, State University of New York. Buffalo, New York 14260, USA

[1] These authors contributed equally to this work.

[*]Correspondence: Cancer Center, Union Hospital, Tongji Medical College, Huazhong University of Science and Technology, Wuhan 430022, China. Fax: +86-27-65650733; Tel:

+86-27-85873100.

Email: Honglin Jin, jin@hust.edu.cn; Kunyu Yang, yangkunyu@hust.edu.cn;

Conflict of interest

The authors have declared that no conflict of interest exists.

Keywords: fibroblasts, CAFs, senescence, radiosensitivity, pulmonary fibrosis, FOXO4-DRI, NSCLC

Supplementary information

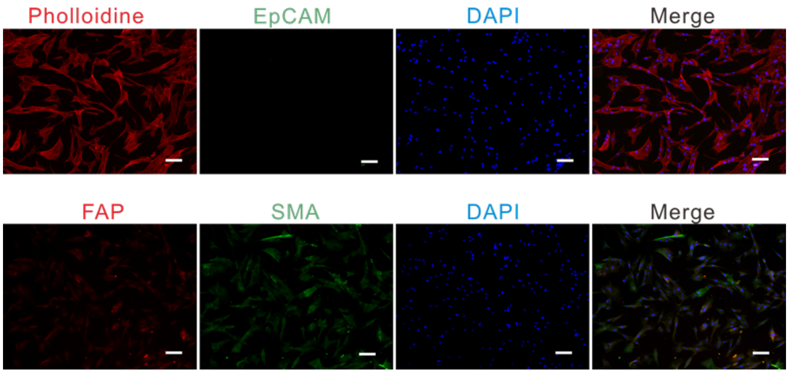

Figure S1. Representative images of primary CAFs obtained from surgical specimens detected by immunofluorescence staining. Scale bar, 100 μm.

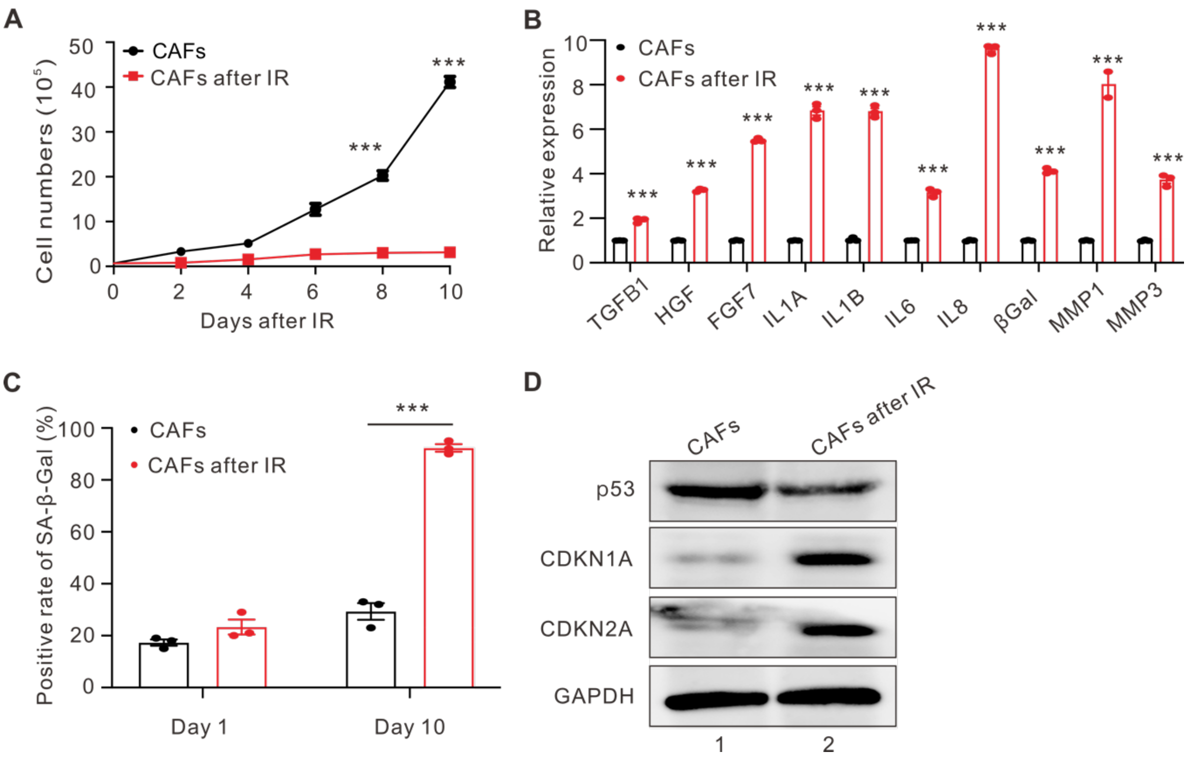

Figure S2. IR-induced CAFs senescence. (A) Cell proliferation curves of CAFs with or without 10-Gy IR. The indicated results represent the means ± SEM of three independent experiments, analyzed by two-way ANOVA. **(B)** mRNA expression of SASP in CAFs 10 days after 10-Gy IR. The indicated results represent the means ± SEM of three independent experiments, analyzed by Student's t test. **(C)** Positive rate of SA-β-Gal staining in CAFs 10 days after 10-Gy IR. The indicated results represent the means ± SEM of three independent experiments, analyzed by two-way ANOVA. **(D)** Western blot identifying the gene expression changes of senescence-associated genes in CAFs 10 days after IR. *** $P < 0.001$.

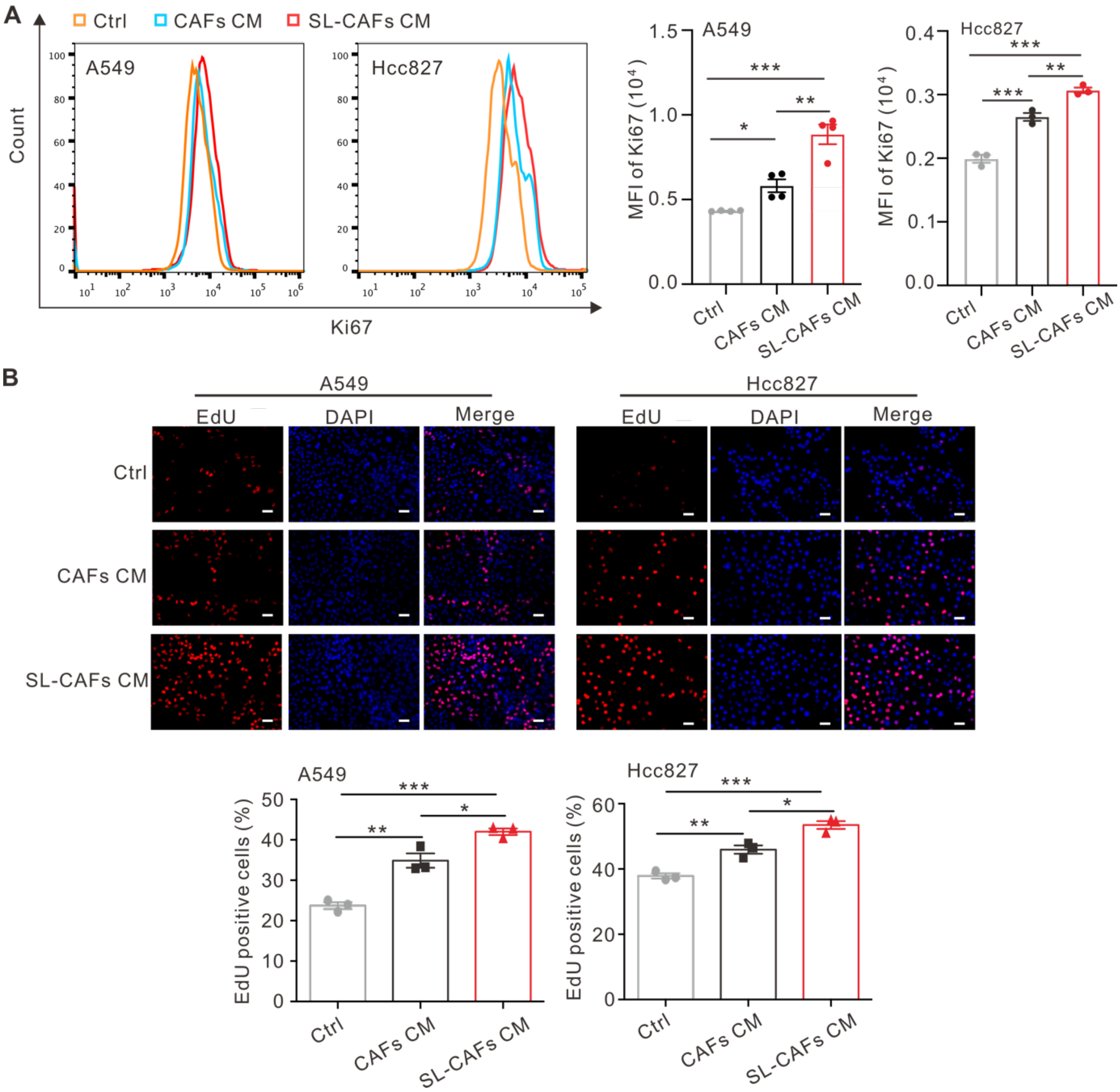

Figure S3. SL-CAFs promote NSCLC cells Ki67 expression. **(A)** Left panel, Ki67 expression of A549 and Hcc827 cells cultured with CAFs CM or SL-CAFs CAFs CM for 48 h detected by flow cytometry. Right panel, quantitative mean fluorescence intensity (MFI) of Ki67 detected by flow cytometry in indicated group of A549 and Hcc827 cells. The results represent the means ± SEM of three or four independent experiments, analyzed by one-way ANOVA. **(B)** Above panel, representative images of EdU staining for A549 and Hcc827 cultured with normal medium, CAFs CM, or SL-CAFs CM for 48 h. Scale bar, 50 μm. Below panel, quantification of EdU positive A549 and Hcc827 cells detected by flow cytometry. The indicated results represent the means ± SEM of three microscopic fields, analyzed by one-way ANOVA. * $P < 0.05$, ** $P <$

0.01, *** $P < 0.001$. SL-CAFs, senescence-like CAFs.

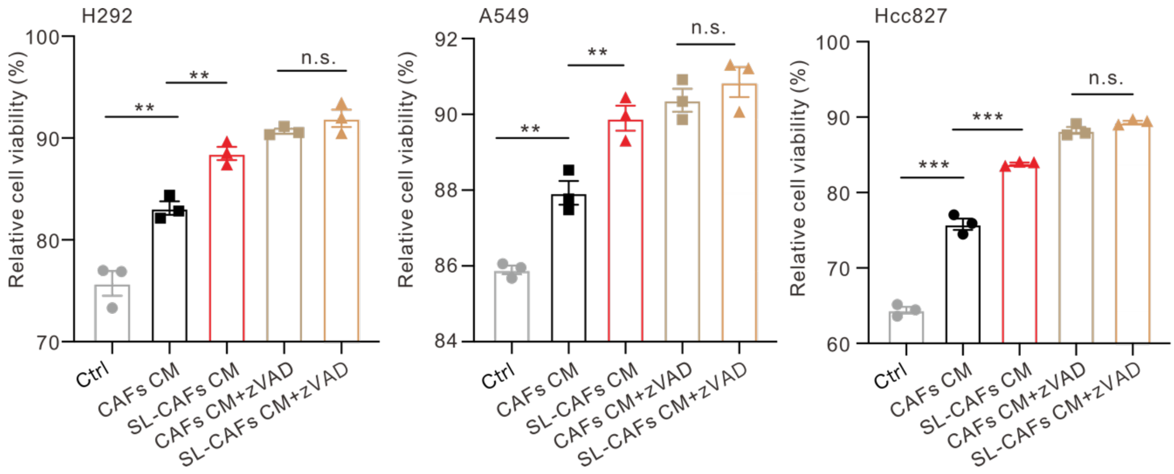

Figure S4. SL-CAFs increases the viability of NSCLC cells by reducing apoptosis after radiation. H292, A549, Hcc827 cells cultured in normal medium, CAFs CM and SL-CAFs CM for 48 h and then irradiated by 8-Gy IR. The cell viability determined 72 h after IR and normalized to non-IR cells by CCK8 assay. The indicated results represent the means ± SEM of three independent experiments, ** $P < 0.01$, *** $P < 0.001$, n.s., not statistically significant analyzed by one-way ANOVA. zVAD, Z-VAD-FMK. SL-CAFs, senescence-like CAFs.

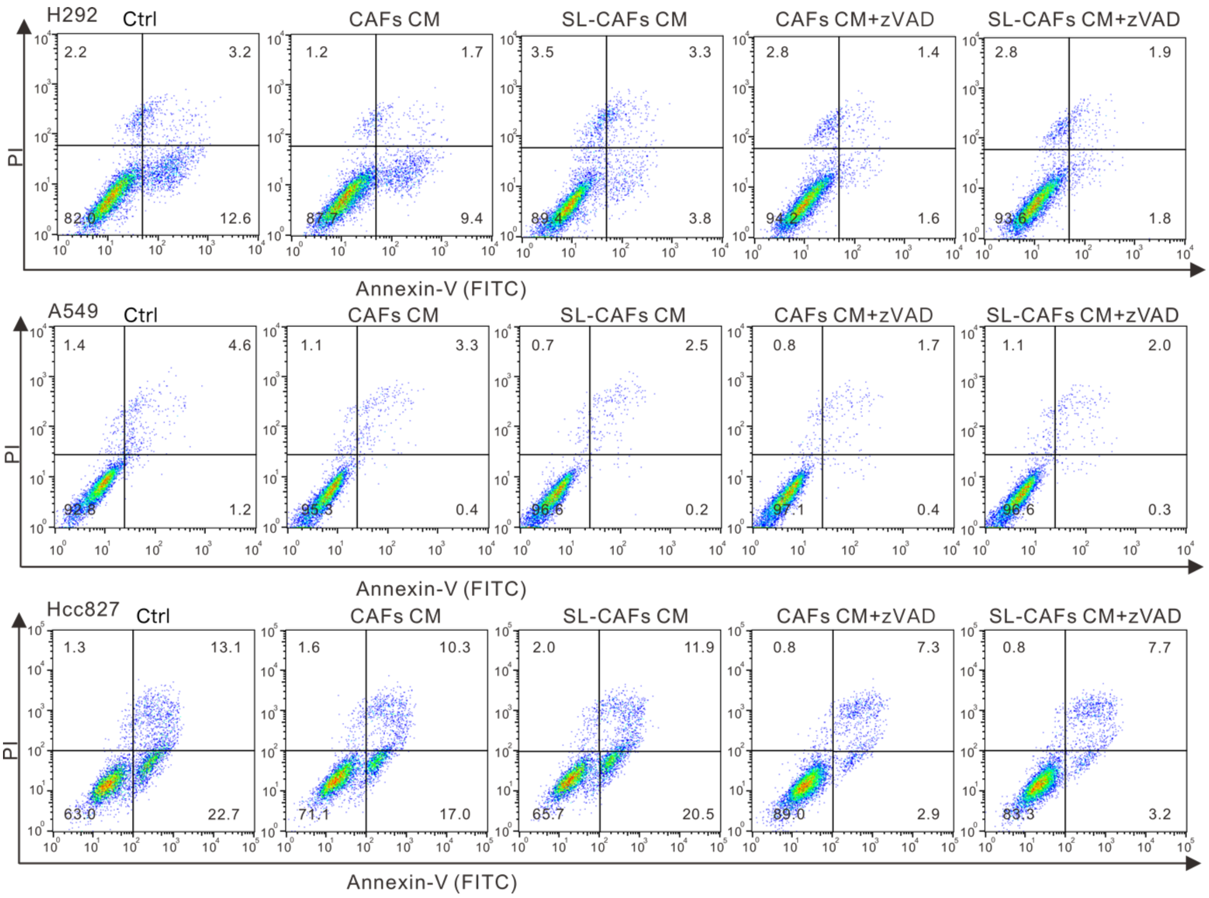

Figure S5. NSCLC cells apoptosis rates after 8-Gy IR detected by flow cytometry. Representative images of H292, A549 and Hcc827 cells apoptosis 72 h after 8-Gy IR detected by flow cytometry using annexin-Ⅴstaining as indicated in different groups. SL-CAFs, senescence-like CAFs.

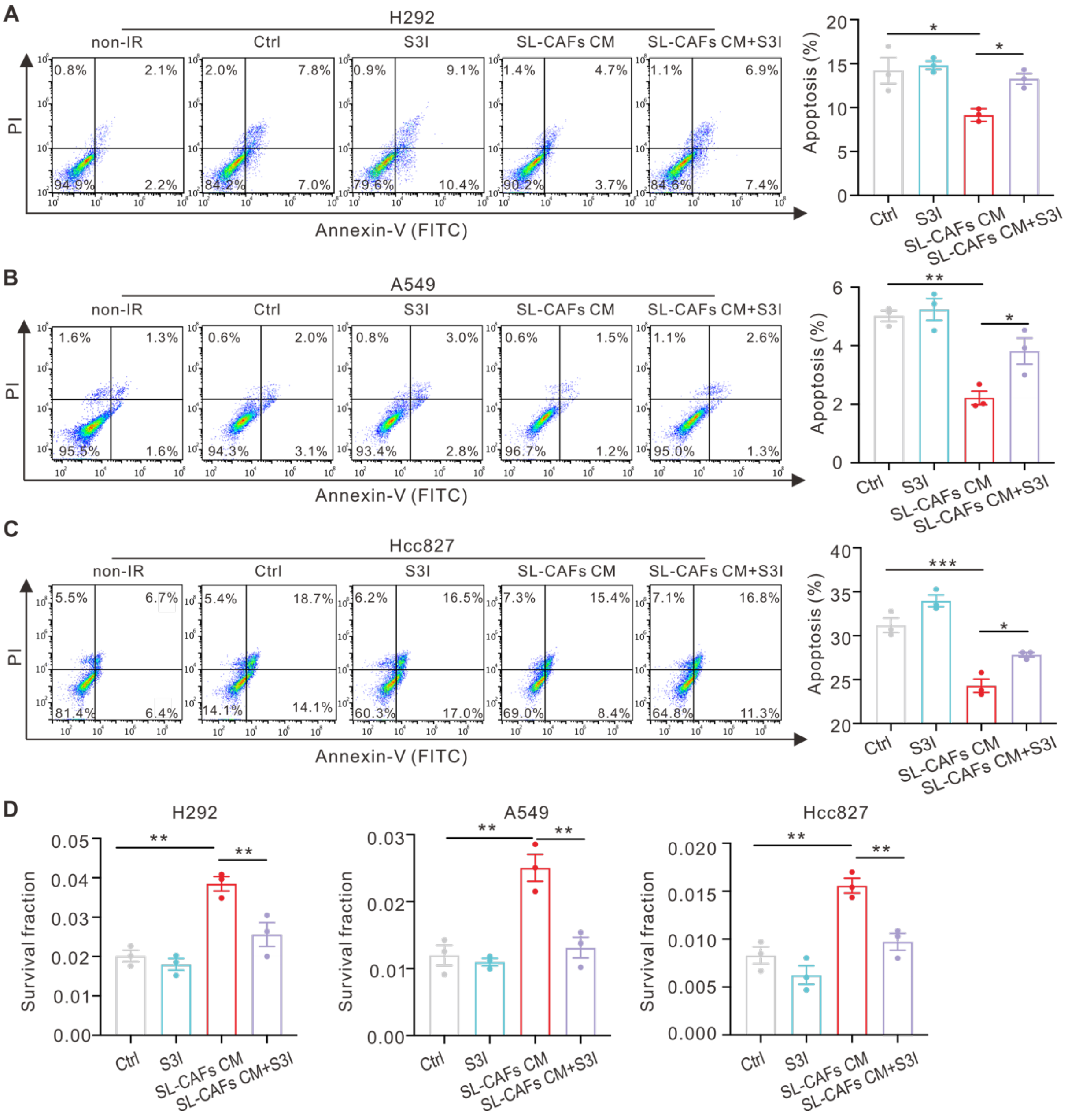

Figure S6. SL-CAFs induce radioresistance of NSCLC cells *via* STAT3 activation. (A-C) H292, A549 and Hcc827 cells apoptosis after 8-Gy IR detected by flow cytometry using annexin-Ⅴ staining (left) and quantitative data of the apoptosis rate (right) as indicated in different groups. **(D)** Clonogenic survival fraction of H292, A549 and Hcc827 cells after 8-Gy IR with STAT3 inhibitor S3I-201. The indicated results represent the means ± SEM of three independent experiments, * $P < 0.05$, ** $P < 0.01$, *** $P < 0.001$, analyzed by one-way ANOVA. S3I, S3I-201. SL-CAFs, senescence-like CAFs.

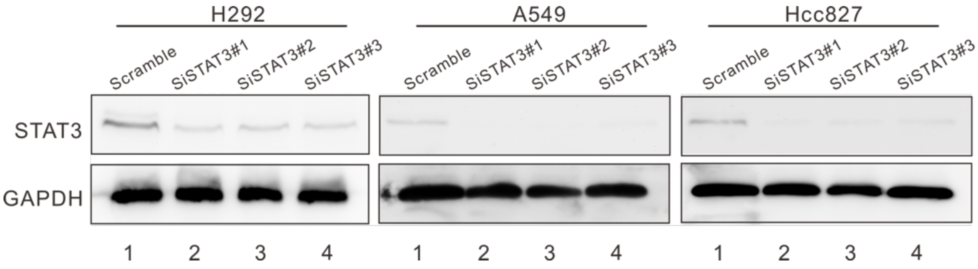

Figure S7. Western blot identifying the knockdown effect of STAT3 gene by three designed siRNA.

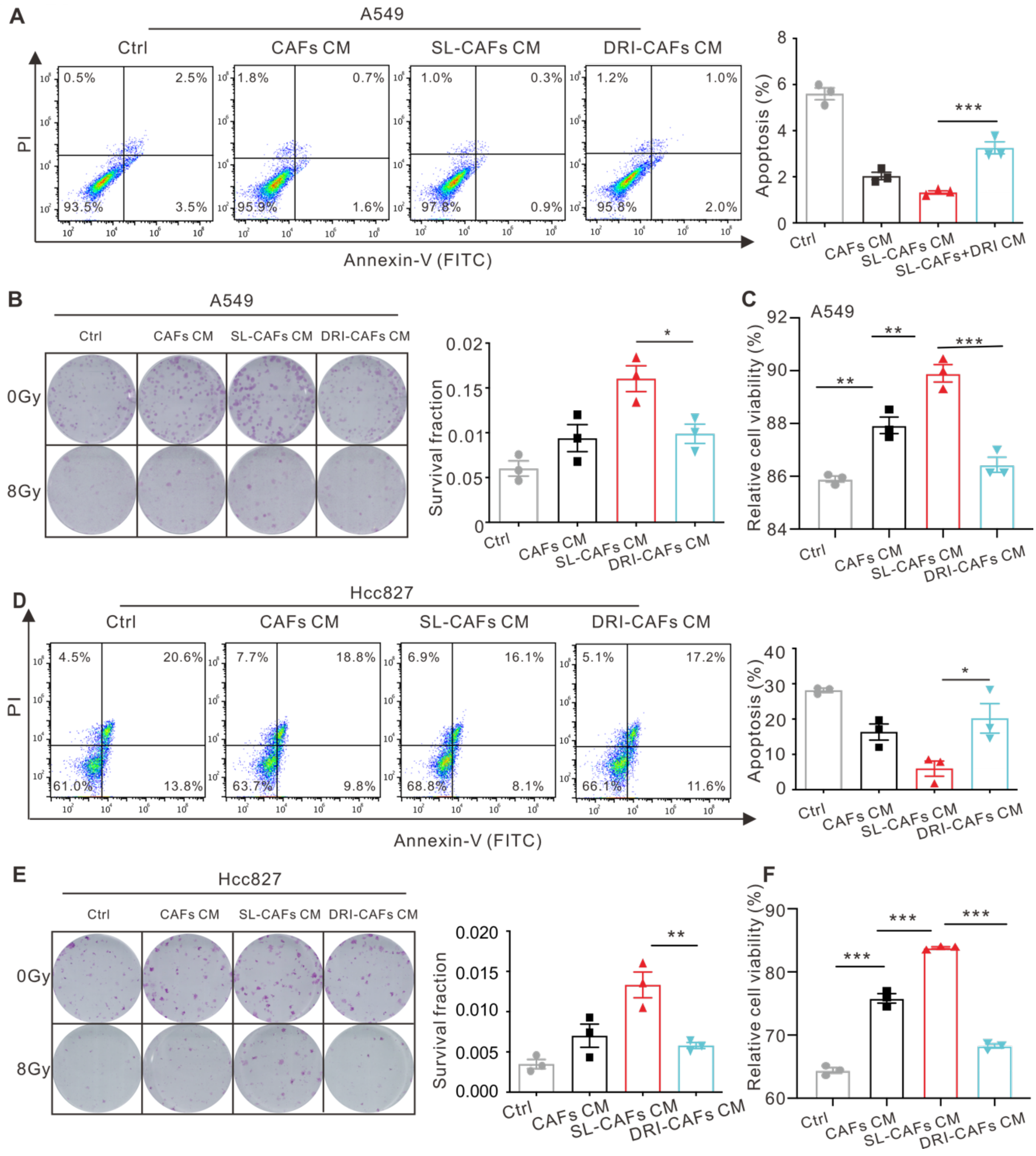

Figure S8. Reverse of A549 and Hcc827 cells radioresistance with FOXO4-DRI by targeting SL-CAFs.

(A) A549 cells apoptosis after 8-Gy IR detected by flow cytometry using annexin-Ⅴstaining (left) and quantitative data of the apoptosis rate (right) as indicated in different groups. **(B)** Left panel, representative images of colony formation in A549 cells cultured in different medium with or without 8-Gy IR; right panel, statistically clonogenic survival fraction of A549 cells with different treatment after 8-Gy IR determined by plate clone formation assay. **(C)** A549 cells cultured in different medium for 48 h and then irradiated by 8-Gy IR. The cell viability determined 72 h after IR by CCK8 assay and normalized to non-IR cells. **(D)** Hcc827 cells apoptosis after 8-Gy IR detected by flow cytometry using annexin-Ⅴstaining (left) and quantitative data of the apoptosis rate (right) as indicated in different groups. **(E)** Left panel, representative images of colony formation in Hcc827 cells cultured in different medium with or without 8-Gy IR; right panel, statistically clonogenic survival fraction of Hcc827 cells with different treatment after 8-Gy IR determined by plate clone formation assay. **(F)** Hcc827 cells cultured in different medium for 48 h and then irradiated by 8-Gy IR. The cell viability determined 72 h after IR by CCK8 assay and normalized to non-IR cells. All the indicated results represent the means ± SEM of three independent experiments, analyzed by one-way ANOVA. *P < 0.05, ** P < 0.01, *** P < 0.001. SL-CAFs, senescence-like CAFs. DRI-CAFs, senescence-like CAFs co-cultured with FOXO4-DRI.

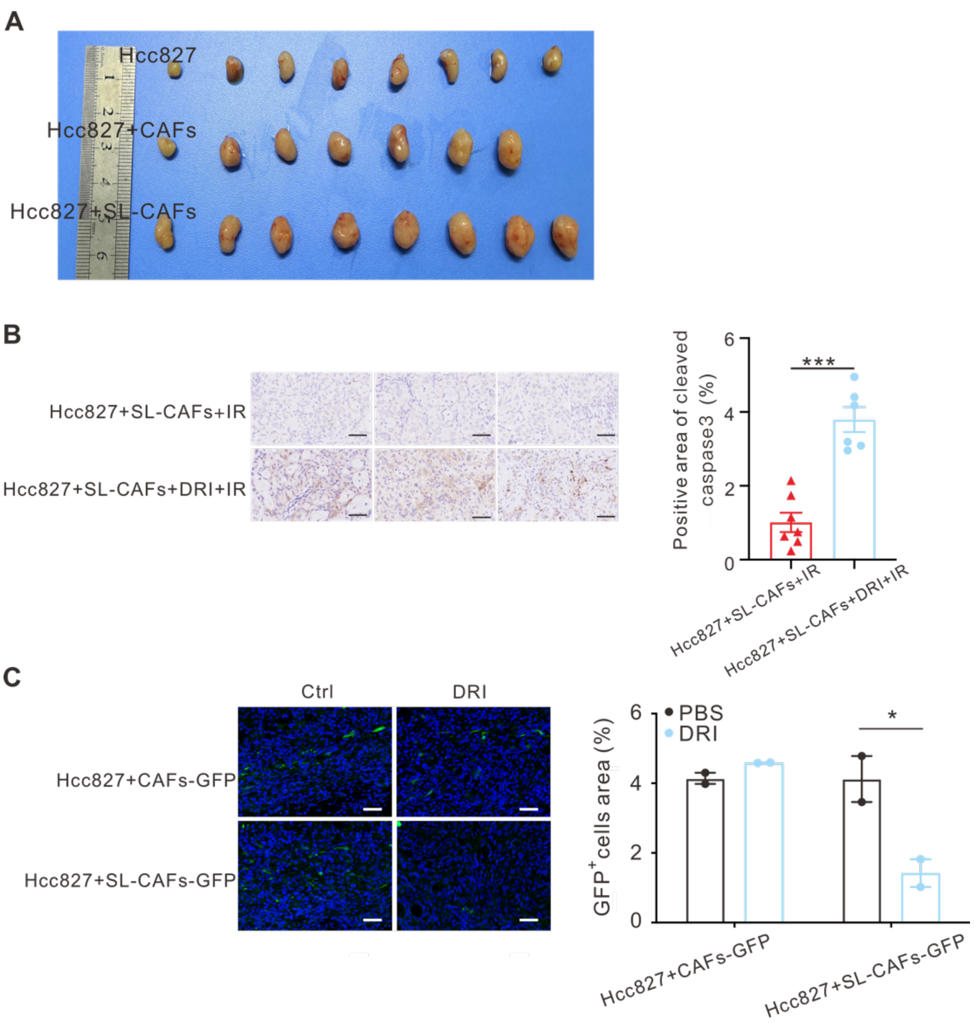

Figure S9. (A) The photo of dissected tumors in mice subcutaneously co-injected of Hcc827 cells with CAFs or SL-CAFs. Hcc827 cells injected alone as control. **(B)** Left panel, representative images of cleaved caspase3 positive cells in xenograft tumors detected by immunohistochemistry staining. Scale bar, 50μm. Right panel, the percentage of cleaved caspase3 positive area in xenograft tumors detected by immunohistochemistry staining. Mean ± SEM (3 microscopic fields at 400× magnification per mice to evaluate the averaged positive area, n = 6-7 mice per group), analyzed by Student's t-test. **(C)**Left panel, representative images of GFP^+ cells in xenograft tumors detected by immunofluorescence staining. Scale bar, 50 μm. Right panel, the percentage of GFP^+ cells area in xenograft tumors detected by immunofluorescence staining. Mean ± SEM (3 microscopic fields at 400× magnification per mice to evaluate

the averaged GFP$^+$ area, n = 2 mice per group), analyzed by Student's t-test. *P < 0.05, *** P < 0.001. SL-CAFs, senescence-like CAFs.

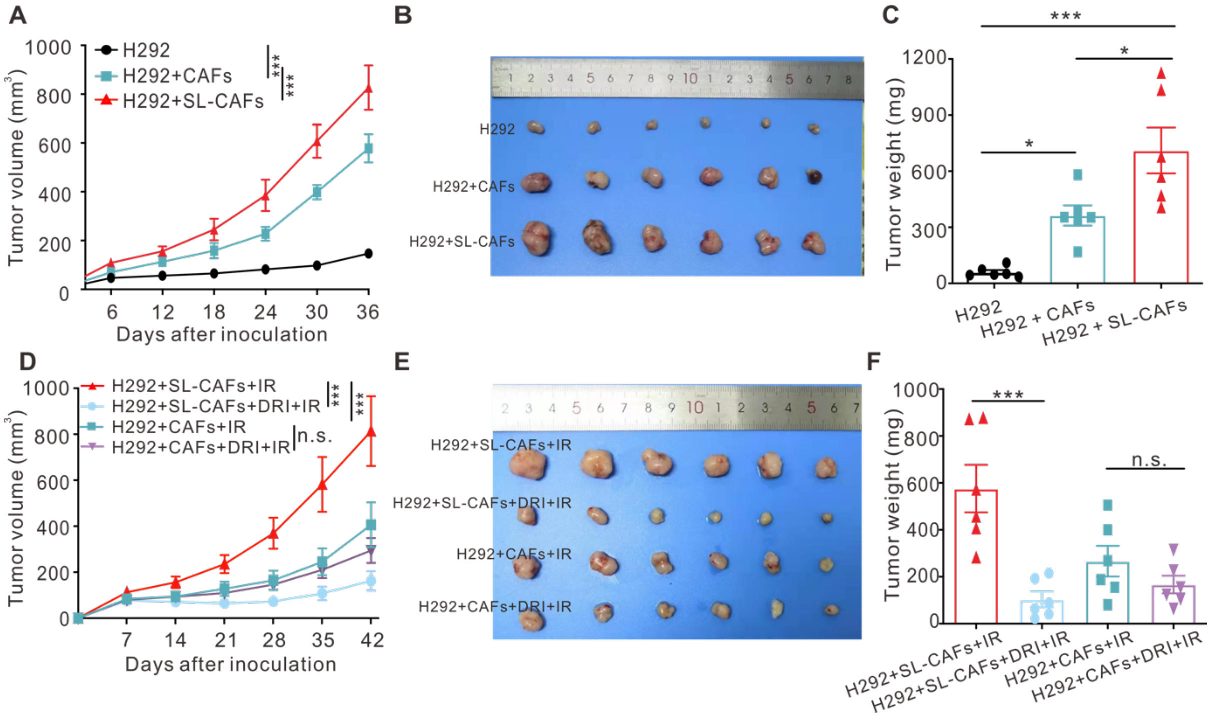

Figure S10. Effect of FOXO4-DRI in improving radiosensitivity *in vivo*. (A) Tumor growth curves for mice subcutaneously co-injected of H292 cells with SL-CAFs or CAFs. H292 cells injected alone as control. Mean ± SEM (n = 6 mice per group), analyzed by two-way ANOVA. **(B)** The photo of dissected tumors in mice subcutaneously co-injected of H292 cells with CAFs or SL-CAFs and **(C)** quantification of tumor weights in each group. Mean ± SEM (n = 6 mice per group), analyzed by one-way ANOVA. **(D)** Tumor growth curves for mice in different groups after IR. Mean ± SEM (n = 6 mice per group), analyzed by two-way ANOVA. **(E)** The photo of dissected tumors and **(F)** quantification of tumor weights in each group after IR. Mean ± SEM (n = 6 mice per group), analyzed by one-way ANOVA. *P < 0.05, *** P < 0.001, n.s., not statistically significant. SL-CAFs, senescence-like CAFs.

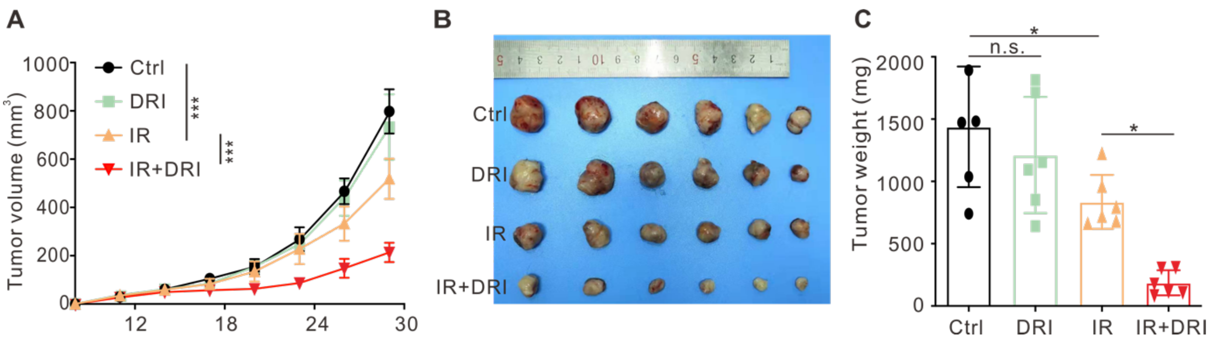

Figure S11. Effect of FOXO4-DRI on improving radiosensitivity *in vivo*. (A) Tumor growth curves for C57BL/6 mice subcutaneously injected of Lewis cells with different treatment. Mean ± SEM (n = 6 mice per group), analyzed by two-way ANOVA. **(B)** The photo of dissected tumors and **(C)** quantification of tumor weights in each group. Mean ± SEM (n = 6 mice per group), analyzed by one-way ANOVA. * $P < 0.05$, *** $P < 0.001$, n.s., not statistically significant.

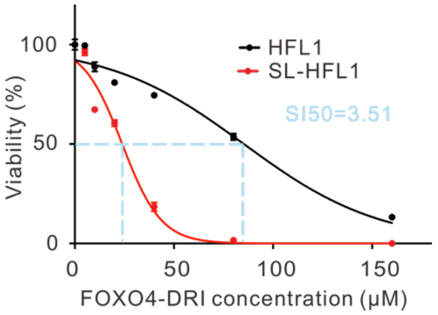

Figure S12. Cell viability assay of HFL1 and SL-HFL1 treating with different concentrations of FOXO4-DRI for 72 h. The indicated results represent the means ± SEM of three independent experiments. SL-HFL1, senescence-like HFL1.

Table S1. Information of patients whose clinical samples were used for isolation of CAFs.

Patients ID	Gender	Age (y)	Pathological type	Stage	TNM
NSCLC001	M	77	LUAD	ⅢA	$T_{1b}N_2M_0$
NSCLC002	M	70	LUAD	ⅠB	$T_{2a}N_0M_0$
NSCLC003	M	71	LUSC	ⅡB	$T_3N_0M_0$
NSCLC004	F	75	LUAD	ⅢA	$T_{1a}N_2M_0$
NSCLC005	M	58	LUAD	ⅠA	$T_{1b}N_0M_0$
NSCLC006	F	48	LUAD	ⅢA	$T_{1c}N_2M_0$
NSCLC007	M	65	LUAD	ⅠA	$T_{1b}N_0M_0$
NSCLC008	F	56	LUAD	ⅢA	$T_4N_0M_0$
NSCLC009	F	55	LUAD	ⅢA	$T_{2a}N_2M_0$
NSCLC010	M	50	LUSC	ⅡB	$T_{2b}N_1M_0$
NSCLC011	F	58	LUAD	ⅠA	$T_{1c}N_0M_0$
NSCLC012	M	65	LUAD	ⅠB	$T_{2a}N_0M_0$
NSCLC013	M	64	LUSC	ⅡA	$T_{2b}N_0M_0$
NSCLC014	M	60	LUSC	ⅡA	$T_{2a}N_1M_0$
NSCLC015	M	62	LUAD	ⅢA	$T_{2a}N_2M_0$
NSCLC016	F	63	LUAD	ⅠA	$T_{1c}N_0M_0$
NSCLC017	M	54	LUAD	ⅠA	$T_{1c}N_0M_0$
NSCLC018	M	67	LUAD	ⅠA	$T_{1b}N_0M_0$
NSCLC019	M	41	LUAD	ⅢB	$T_4N_2M_0$
NSCLC020	M	57	LUAD	ⅠA	$T_{1c}N_0M_0$
NSCLC021	M	67	LUSC	ⅡA	$T_{2b}N_0M_0$
NSCLC022	F	56	LUAD	ⅠA	$T_{1c}N_0M_0$
NSCLC023	M	61	LUAD	ⅠB	$T_{2a}N_0M_0$
NSCLC024	F	61	LUAD	ⅢA	$T_{2a}N_2M_0$

NSCLC = non-small cell lung cancer, M = male, F = female, LUAD = lung adenocarcinoma, LUSC = lung squamous cell carcinoma, T = tumor, N = node, M = metastasis.

Table S2. Plating efficiencies of NSCLC cells in plate clone formation assay.

	H292			A549			Hcc827		
	Ctrl	CAFs CM	SL-CAFs CM	Ctrl	CAFs CM	SL-CAFs CM	Ctrl	CAFs CM	SL-CAFs CM
0 Gy	0.1853	0.1853	0.1907	0.3373	0.3433	0.3327	0.1900	0.1893	c0.1907
2 Gy	0.0623	0.0757	0.0913	0.1183	0.1353	0.1320	0.0530	0.0653	0.0660
4 Gy	0.0255	0.0408	0.0540	0.0438	0.0570	0.0645	0.0093	0.0173	0.0262
6 Gy	0.0073	0.0163	0.0318	0.0107	0.0145	0.0213	0.0015	0.0033	0.0067
8 Gy	0.0009	0.0028	0.0086	0.0018	0.0032	0.0074	0.0003	0.0009	0.0023

Table S3. Sequences of primer used in RT-qPCR analysis.

Gene name	Forward primer	Reverse primer
TP53	GAGTCAACGGATTTGGTCGT	GACAAGCTTCCCGTTCTCAG
CDKN1A	GACAGCAGAGGAAGACCATGTGGAC	GAGTGGTAGAAATCTGTCATGCTG
CDKN2A	CCAACGCACCGAATAGTTACG	GCGCTGCCCATCATCATG
IL1A	AGATGCCTGAGATACCCAAAACC	CCAAGCACACCCAGTAGTCT
IL1B	ATGATGGCTTATTACAGTGGCAA	GTCGGAGATTCGTAGCTGGA
IL6	CGGTCCAGTTGCCTTCTCCC	GAGTGGCTGTCTGTGTGGGG
IL8	CTCTTGGCAGCCTTCCTGATT	TATGCACTGACATCTAAGTTCTTTAGCA
βGal	TTAGGATGTGCATTTTCACCTGA	CTTTGGCACTGCAGGGATG
TGFB1	CAATTCCTGGCGATACCTCAG	GCACAACTCCGGTGACATCAA
FGF7	CTGAGGATCGATAAAAGAGGCAA	ATTCTTCATCTCTTGGGTCCCTT
HGF	GTTCCTGGTCGTGGATGTGC	TCGGACAAAAATACCAGGACG
MMP1	GGGCTTGAAGCTGCTTACGA	TGTCCCTGAACAGCCCAGTAC
MMP3	AGAGGTGACTCCACTCACAT	GGTCTGTGAGTGAGTGATAG

Table S4. Sequences of three designed siRNA targeting STAT3.

SiRNA	Sense（5'-3'）	Anti-sense（3'-5'）
#1	CCACUUUGGUGUUUCAUAATT	UUAUGAAACACCAAAGUGGTT
#2	GCAACAGAUUGCCUGCAUUTT	AAUGCAGGCAAUCUGUUGCTT
#3	CCCGUCAACAAAUUAAGAATT	UUCUUAAUUUGUUGACGGGTT
